# Segmentation methods for quantifying X-ray Computed Tomography based biomarkers to assess hip fracture risk: a systematic literature review

**DOI:** 10.3389/fbioe.2024.1446829

**Published:** 2024-10-23

**Authors:** Cristina Falcinelli, Vee San Cheong, Lotta Maria Ellingsen, Benedikt Helgason

**Affiliations:** ^1^ Department of Engineering and Geology, University “G. D’Annunzio” of Chieti-Pescara, Pescara, Italy; ^2^ Institute for Biomechanics, ETH-Zurich, Zurich, Switzerland; ^3^ Future Health Technologies Programme, Singapore-ETH Centre, CREATE campus, Singapore, Singapore; ^4^ Faculty of Electrical and Computer Engineering, University of Iceland, Reykjavik, Iceland

**Keywords:** segmentation, finite element modeling, hip fracture risk, computed tomography, osteoporosis, CT-derived biomarkers

## Abstract

**Background:**

The success of using bone mineral density and/or FRAX to predict femoral osteoporotic fracture risk is modest since they do not account for mechanical determinants that affect bone fracture risk. Computed Tomography (CT)-based geometric, densitometric, and finite element-derived biomarkers have been developed and used as parameters for assessing fracture risk. However, to quantify these biomarkers, segmentation of CT data is needed. Doing this manually or semi-automatically is labor-intensive, preventing the adoption of these biomarkers into clinical practice. In recent years, fully automated methods for segmenting CT data have started to emerge. Quantifying the accuracy, robustness, reproducibility, and repeatability of these segmentation tools is of major importance for research and the potential translation of CT-based biomarkers into clinical practice.

**Methods:**

A comprehensive literature search was performed in PubMed up to the end of July 2024. Only segmentation methods that were quantitatively validated on human femurs and/or pelvises and on both clinical and non-clinical CT were included. The accuracy, robustness, reproducibility, and repeatability of these segmentation methods were investigated, reporting quantitatively the metrics used to evaluate these aspects of segmentation. The studies included were evaluated for the risk of, and sources of bias, that may affect the results reported.

**Findings:**

A total of 54 studies fulfilled the inclusion criteria. The analysis of the included papers showed that automatic segmentation methods led to accurate results, however, there may exist a need to standardize reporting of accuracy across studies. Few works investigated robustness to allow for detailed conclusions on this aspect. Finally, it seems that the bone segmentation field has only addressed the concept of reproducibility and repeatability to a very limited extent, which entails that most of the studies are at high risk of bias.

**Interpretation:**

Based on the studies analyzed, some recommendations for future studies are made for advancing the development of a standardized segmentation protocol. Moreover, standardized metrics are proposed to evaluate accuracy, robustness, reproducibility, and repeatability of segmentation methods, to ease comparison between different approaches.

## 1 Introduction

Hip fractures account for significantly higher disability, mortality and socio-economic costs compared to other skeletal fractures. Approximately 20-25% of elderly patients die within 6-months post-fracture and the majority of survivors do not return to their pre-fracture state (Haleem et al., 2008). Therefore, effective early screening of patients at risk of developing a fragility fracture is important, as hip fracture incidence is expected to increase to 6.3 million in 2050, in the aging North American, European, and other industrialized countries' populations. Notably, it is projected that more than 50% of hip fractures will occur in Asia by the year 2050 (Cong and Walker, 2014).

A major contributor to elevated fracture risk at the hip is osteoporosis, affecting mostly the elderly population. The current clinical standard for diagnosing osteoporosis is the use of areal bone mineral density (aBMD) derived from dual-energy X-ray absorptiometry (DXA) scans, with an aBMD of more than 2.5 standard deviations lower than the mean of the healthy young adult female (T-score ≤ -2.5) as the threshold for a positive diagnosis (Kanis et al., 2008). However, aBMD lacks both sensitivity and specificity, as around 50% of incident fractures occur in individuals who do not have osteoporosis at the time when the scan is acquired and around 50% of individuals diagnosed with osteoporosis will not sustain a hip fracture during a study’s follow-up period (Schuit et al., 2004; Stone et al., 2003; Wainwright et al., 2005). Another fracture risk assessment tool, which is a part of the clinical guidelines in many countries, is the FRAX-score, used with or without aBMD (https://frax.shef.ac.uk/FRAX/). However, the performance of FRAX in identifying cases at high risk of fracture compared to aBMD is controversial and depends on the intervention threshold. In some studies, FRAX was found to be sensitive in terms of identifying subjects at risk of sustaining a major osteoporosis fracture, whereas an opposite result was found for some studies (Jiang et al., 2017).

The current standards for assessing hip fracture risk do not directly include information on material and structural determinants, such as bone and soft tissue geometry, mechanical properties, and loading, that are factors known to affect bone fracture risk (Bouxsein, 2005; Keaveny et al., 2020). For these reasons, there exists a need for developing more accurate image-based biomarkers for quantifying hip fracture risk that take some or all these factors into account. To this end, Computed Tomography (CT)-based methods have been developed to derive geometric and densitometric biomarkers, such as cortical thickness (Treece et al., 2015), volumetric bone mineral density (vBMD) (Black et al., 2008), bone mass (Treece et al., 2015), and bone volume (Cheng et al., 2007). The predictive power of CT-based biomarkers has been quantified in several studies (Table 1), demonstrating a significant association with hip fracture risk. However, in most studies, CT-derived measures alone or in combination with other markers, did not classify fractures significantly better than DXA-derived aBMD (Black et al., 2008; Chalhoub et al., 2016; Cheng et al., 2007). In contrast, CT-based finite element (FE)-derived biomarkers enable more accurate representation of heterogeneous distribution of bone density and strength based on the bone geometry. CT-based subject-specific FE models have been studied extensively and demonstrated to accurately predict the mechanical response of the proximal femur under loading (Bessho et al., 2007; Dall’Ara et al., 2013; Dragomir-Daescu et al., 2011; Duchemin et al., 2008; Grassi et al., 2012; Keyak, 2001; Keyak et al., 2005; Koivumäki et al., 2012; Nishiyama et al., 2013; Schileo et al., 2020; 2014; 2007; Varga et al., 2016; Yosibash et al., 2014) and the response of the whole hip region under simulated impact (Fleps et al., 2019). There exists ample evidence to suggest that most hip fractures, in vulnerable populations at least, are the result of a fall from standing height or lower (Hayes et al., 1996; Parkkari et al., 1999; Scott et al., 2010). As such, FE-derived strength or load-to-strength ratio computed through CT-based subject-specific FE models have been used as parameters for assessing osteoporotic hip fracture risk (Table 2). However, the improvement over DXA-based aBMD in predicting hip fracture risk is not uniform and depends on the patient cohort analyzed. When tested on pre-fracture cohorts, most studies found that FE-derived predictors performed equivalently to DXA-derived aBMD in classifying incidence hip fractures with the exception of two studies. Fleps et al. (2022) found FE models to be a better classifier than aBMD in the AGES Reykjavik study cohort. Moreover, Yosibash et al. (2023) showed that 7/11 of subjects that had DXA imaging who subsequently fractured had non-osteoporotic aBMD score. When post-fracture CT images have been used, CT-based FE strength estimates performed significantly better than aBMD in classifying fracture cases (Bhattacharya et al., 2019; Falcinelli et al., 2014; Qasim et al., 2016).

**TABLE 1:** Literature overview of CT-derived densitometric (plain text) and geometric biomarkers (italic) used to classify osteoporotic hip fractures.

Reference	Study	Type of imaging	Gender (F or M) Subjects (N) Cases (Fx)	CT-based biomarker	Performance
Cheng et al. (2007) ^,^ ^a^	Age-matched case-control study	Post fracture imaging	F; N=111, Fx=45	FN, TR and Total vBMD-I, vBMD-T, vBMD-C; *integral tissue volumes* (FN, TR and total femurs); *cortical tissue volumes* (FN, TR and total femurs); *CSAs* (*min at FN, max at TR*); *strength indices* (*NBSI, NCSI, TCSI*); FN *axis length*; *cortical volume/total volume*; *iCthi*; *BR*	AUC=0.87 for FN vBMD-I AUC=0.80 for FN vBMD-T AUC=0.80 for FN vBMD-C AUC=0.84 for FN aBMD AUC=0.86 for TR vBMD-I AUC=0.88 for TR vBMD-T AUC=0.80 for TR vBMD-C AUC=0.84 for TR aBMD AUC=0.87 for Total vBMD-I AUC=0.88 for Total vBMD-T AUC=0.81 for Total vBMD-C AUC=0.88 for Total aBMD AUC=0.76 for *integral tissue volume FN* AUC=0.78 for *integral tissue volume TR* AUC=0.78 for *integral tissue volume total* femur AUC=0.82 for *cortical tissue volume FN* AUC=0.82 for *cortical tissue volume TR* AUC=0.81 for *cortical tissue volume total* femur AUC=0.79 for *CSA min* AUC=0.79 for *CSA max* AUC=0.76 for *NBSI* AUC=0.84 for *NCSI* AUC=0.89 for *TCSI* AUC=0.79 for FN *axis length* AUC=0.855 for *cortical volume/total volume* AUC=0.864 for *iCthi* AUC=0.856 for *BR*
Black et al. (2008)	Prospective cross-sectional MrOS	Pre fracture imaging	M; N=3347, Fx=42	vBMD-I; vBMD-C; vBMD-T; *%CV*; *minimum CSA* in FN	AUC=0.855 combining CT parameters AUC=0.853 for aBMD from DXA
Ito et al. (2010)	Two age-matched case-control studies	Post fracture imaging	F; N=40, Fx=20 F; N=32, Fx=16	*Hip axis length*; *CSMI*; *BR*; *NSA*; *CSA*	Study 1: OR=2.15 pvalue=0.07 for *hip axis length* OR=1.52 pvalue=0.06 for *CSMI* OR=2.56 pvalue=0.01 for *BR* Study 2: OR=2.15 pvalue=0.11 for *NSA* OR=1.47 pvalue=0.01 for *cortical CSA*
Johannesdottir et al. (2011) ^,^ ^a^	Case-control study nested within the prospective study AGES	Pre fracture imaging	F; N=275, Fx=88 M; N=166, Fx=55	*Cth* at the mid-FN in anatomical quadrants; vBMD	F: HR=1.8 for SA *Cth* (any hip fracture) HR=1.8 for SA *Cth* (FN fracture) HR=2.1 for SA *Cth* (trochanteric fracture) HR=1.9 for vBMD (any hip fracture) HR=1.8 for vBMD (FN fracture) HR=2.4 for vBMD (trochanteric fracture) HR=1.8 for aBMD (any hip fracture) HR=1.7 for aBMD (FN fracture) HR=2.1 for aBMD (trochanteric fracture) M: HR=3.6 for SA *Cth* (any hip fracture) HR=3.5 for SA *Cth* (FN fracture) HR=4.3 for SA *Cth* (trochanteric fracture) HR=2.9 for vBMD (any hip fracture) HR=2.9 for vBMD (FN fracture) HR=3.2 for vBMD (trochanteric fracture) HR=3.1 for aBMD (any hip fracture) HR=2.7 for aBMD (FN fracture) HR=4.4 for aBMD (trochanteric fracture)
Bousson et al. (2011)	Prospective EFFECT	Post fracture imaging	F; N=107, Fx=47	vBMD-I FH; vBMD-T TR; *CortShaftThick*; *CortNeckThick*	AUC=0.821 for vBMD-I FH + *CortShaftThick* AUC=0.819 for vBMD-I FH + *CortNeckThick* AUC=0.803 for vBMD-T TR + *CortShaftThick* AUC=0.777 for aBMD from DXA
Yang et al. (2012)	Prospective MrOS	Pre fracture imaging	M; N=250 Fx=40	vBMD-I, vBMD-C, vBMD-T, *CTh*, *CSA* in different quadrants of FN, IT and TR	AUC=0.675 for vBMD-C at the inferomedial FN; AUC=0.88 for vBMD-T at superolateral FN, medial IT and medial TR; AUC=0.896 for age + vBMD-T at the superolateral FN and medial TR; AUC=0.829 for TH aBMD; AUC=0.863 TH aBMD + age; AUC=0.901 age + vBMD-T at the superolateral FN and medial TR + TH aBMD
Bredbenner et al. (2014)	Prospective MrOS	Pre fracture imaging	M; N=450 Fx=40	SSDM	AUC=0.94 for SSDM AUC= 0.94 for SSDM + age AUC=0.93 for SSDM + age + aBMD AUC=0.82 for TH aBMD AUC=0.83 for aBMD + age AUC=0.83 for aBMD + age + BMI
Treece et al. (2015)	Prospective MrOS	Pre fracture imaging	M; N=407 Fx=99	FN, TH and trochanteric vBMD-I, vBMD-C, vBMD-T; CM and ECTD from CBM	All fractures: AUC=0.76 for vBMD + age+site+height AUC=0.79 for CBM+age+site+height AUC=0.78 for aBMD+age+site+height Femoral neck fractures: AUC=0.73 for vBMD + age+site+height AUC=0.82 for CBM+age+site+height AUC=0.76 for aBMD+age+site+height Trochanteric fractures: AUC=0.73 for vBMD + age+site+height AUC=0.78 for CBM+age+site+height AUC=0.71 for aBMD+age+site+height
Chalhoub et al. (2016)	Prospective MrOS	Pre fracture imaging	M; N=3302, Fx=119 (hip fractures)	FN vBMD-C, FN vBMD-T, TH vBMD-C, TH vBMD-T	Hip fractures: AUC=0.69 for FN vBMD-C AUC=0.72 for FN vBMD-T AUC=0.76 for FN aBMD from DXA
Borggrefe et al. (2016)	Prospective MrOS	Pre fracture imaging	M; N=230, Fx=65	FN vBMD, TR vBMD, TH vBMD, *FN BR*, *TR BR*, *FN LTI*, *TR LTI*, *FN Zmin*, *TR Zmin*	HC=0.81 for TH vBDM HC=0.78 for FN vBMD HC=0.82 for TH vBMD+ *FN BR*, *FN Zmin* HC=0.81 for TH aBMD HC=0.82 for TH aBMD + *FN BR*
Museyko et al. (2016)	Prospective EFFECT	Post fracture imaging	F; N=102, Fx=46	*SL BR*; *SL CortArea*; SL vBMD-T; *CTh*; vBMD-I	All models were adjusted for age, height, and weight: AUC=0.82 for SL vBMD-T SA + SL CortArea SP AUC=0.83 for SL vBMD-T SA + SL CortArea SP + SL BR AUC=0.83 for SL vBMD-T SA + SL CortArea SP + FN CTh All AUC=0.83 for SL vBMD-T SA + SL CortArea SP +FH vBMD-I IA AUC=0.83 for SL vBMD-T SA + SL CortArea SP + FN CTh SP AUC=0.86 for TH vBMD-I + SL vBMD-T SA SL CortArea SP AUC=0.86 for TH vBMD-I + SL vBMD-T SA SL CortArea SP + SL BR AUC=0.86 for TH vBMD-I + SL vBMD-T SA SL CortArea SP + FN CTh All AUC=0.77 for TH vBMD-I AUC=0.83 for TR vBMD-T + FN CTh All AUC=0.88 for TR vBMD-T + FN CTh All + SL vBMD-T SA + SL CortArea SP AUC=0.88 for TR vBMD-T + FN CTh All + SL vBMD-T SA + SL CortArea SP + SL BR Comparison with aBMD NR
Khoo et al. (2020) ^,^ ^a^	Case-control study	Post fracture imaging	F; N=546, Fx=285	*FN Delta*, FN Sigma	AUC=0.87 for age, weight, height, FN aBMD, *FN Delta*, and FN Sigma AUC=0.84 for age, weight, height, and FN aBMD
Wang et al. (2022)	Cross-sectional case-control study	Post fracture imaging	F; N=562, Fx=236	*TH CTh*; *IT CTh*; *FH V*; *THRCTM*; *FN CSA*	All models were adjusted for age, height and weight: AUC=0.805 for *TH CTh* + *FH Vol* + *THRCTM* + *FN CSA* AUC=0.728 for *THCortThick* + *FH Vol*+*FN CSA* AUC=0.735 for *IT CTh + FH Vol* + *FN CSA* AUC=0.735 for *IT CTh* + *FH Vol* AUC= 0.703 for *IT CTh* + *FN CSA*

^a^ DXA was not used in this study, CT was also used to measure a DXA-equivalent hip aBMD

**TABLE 2:** CT-based FE model-derived biomarkers used to classify hip fractures

Reference	Study	Type of imaging	Gender (F or M) Subjects (N) Fractured cases (Fx)	Types of CT-based biomarkers	Performance
Orwoll et al. (2009)	Prospective MrOS	Pre fracture imaging	M; N=250 Fx=40	FE-strength, load-to-strength ratio	AUC=0.83 for FE strength AUC=0.79 for load-to-strength ratio AUC=0.85 for aBMD AUC=0.87 for FE strength +age + BMI + clinical center AUC=0.88 for load-to-strength ratio + age + BMI+ clinical center AUC=0.88 for aBMD +age + BMI + clinical center
Amin et al. (2011)	Case-control study	Pre fracture imaging	F; N=314, Fx=55 M; N=266, Fx=28	FE-strength, load-to-strength ratio	F: AUC=0.84 for FE strength AUC=0.84 for load-to-strength ratio AUC=0.85 for TH vBMD AUC=0.84 for TH aBMD M: AUC=0.78 for FE strength AUC=0.77 for load-to-strength ratio AUC=0.78 for TH vBMD AUC=0.78 for TH aBMD
Kopperdahl et al. (2014) ^,^ ^a^	Prospective AGES	Pre fracture imaging	F; N=608, Fx=108 M; N=440, Fx=63	FE strength, load-to-strength ratio	AUC=0.78 for FE strength (female) AUC=0.84 for FE strength (male) AUC=0.80 for FE strength+age (female) AUC=0.86 for FE strength+age (male)
Nishiyama et al. (2014)	Case-control study	Post fracture imaging	F; N=70, Fx=35	FE strength, vBMD	Pooled fractures: AUC=0.87 for vBMD AUC=0.89 for FE strength AUC=0.94 for vBMD+FE strength Neck Fractures: AUC=0.86 for vBMD AUC=0.94 for FE strength AUC=0.94 for vBMD+FE strength Trochanteric fractures: AUC=0.83 for vBMD AUC=0.79 for FE strength AUC=0.86 for vBMD+FE strength
Falcinelli et al. (2014)	Case-control study	Post fracture imaging	F; N=55, Fx=22	FE strength	AUC=0.87 for FE strength in stance AUC=0.88 for FE strength in fall AUC=0.73 for FN aBMD AUC=0.79 for TH aBMD AUC=0.75 for trochanteric aBMD
Qasim et al. (2016)	Retrospective study	Post fracture imaging	F; N=100, Fx=50	FE strength	AUC=0.75 for FE strength in stance AUC=0.79 for FE strength in fall AUC=0.75 for FN aBMD AUC=0.74 for TH aBMD AUC=0.79 for FE strength in stance + aBMD AUC=0.80 for FE strength in fall + aBMD
Adams et al. (2018)	Retrospective case-cohort study preexisting FOCUS	Pre fracture imaging	F; N=850 M; N=465	FE strength	F: AUC=0.73 for FE strength AUC=0.72 for vBMD AUC=0.72 for aBMD M: AUC=0.75 for FE strength AUC=0.71 for vBMD AUC=0.73 for aBMD
Bhattacharya et al. (2019)	Retrospective study	Post fracture imaging	F; N=98, Fx=49	ARF0, FE strength	AUC=0.85 for ARF0 AUC=0.82 for FE strength AUC=0.75 for aBMD
Enns-Bray et al. (2019) ^,^ ^a^	Prospective AGES	Pre fracture imaging	F; N=254, Fx=95	FE strain+fall probability	AUC=0.73 for FE strain+fall AUC=0.70 for aBMD
Michalski et al. (2021)	Prospective study	Pre fracture imaging	F; N=187, Fx=66 M; N=303, Fx=57	TH vBMD-I, FE strength	Pooled: AUC=0.661 for TH vBMD-I AUC=0.675 for FE strength AUC=0.675 for FE strength+TH vBMD-I F: AUC=0.664 for TH vBMD-I AUC=0.679 for FE strength AUC=0.693 for FE strength+TH vBMD-I M: AUC=0.65 for TH vBMD-I AUC=0.618 for FE strength AUC=0.644 for FE strength+TH vBMD-I Performance of DXA-based aBMD NR
Fleps et al. (2022) ^,^ ^a^	Prospective AGES	Pre fracture imaging	F; N=362, Fx=142 M; N=239, Fx=59	FE strength	F: AUC=0.74 for FE strength AUC=0.69 for aBMD M: AUC=0.78 for FE strength AUC=0.72 for aBMD
Cao et al. (2022) ^,^ ^a^	Prospective AGES	Pre fracture imaging	F; N=211, Fx=68 M; N=134, Fx=42	FE ultimate strength, FE yield strength, FE energy to failure, PC1	Whole: AUC= 0.699 for aBMD + covariates^b^ AUC=0.738 for PC1 + aBMD + covariates^b^ AUC= 0.724 for FE parameters combined, aBMD + covariates^b^ AUC=0.754 for PC1 + aBMD +covariates^b^ AUC=0.651 for FRAX F: AUC= 0.608 for aBMD + covariates^b^ AUC=0.623 for PC1 + aBMD + covariates^b^ AUC= 0.669 for FE parameters combined, aBMD + covariates^b^ AUC=0.71 for PC1 + aBMD +covariates^b^ AUC=0.623 for FRAX M: AUC= 0.727 for aBMD + covariates^b^ AUC=0.745 for PC1 + aBMD + covariates^b^ AUC= 0.724 for FE parameters combined, aBMD + covariates^b^ AUC=0.825 for PC1 + aBMD +covariates^b^ AUC=0.705 for FRAX

^a^ DXA was not used in this study, CT was also used to measure a DXA-equivalent hip aBMD

^b^ Covariates: age, sex, height, weight, health status, and bone medication status

Although CT-derived biomarkers for assessing hip fracture risk have shown good potential for improving the performance of aBMD and T-score (Tables 1 and 2), the largest studies on CT-derived biomarkers are a couple of orders of magnitudes smaller in terms of the number of subjects, than the largest studies on FRAX and aBMD. This is related to the fact that to quantify many of the CT-based biomarkers, segmentation of the bones in the hip from the CT data is needed. When the segmentation is done manually or semi-automatically, which is known to be labor intensive and expensive, this essentially prevents these biomarkers from being adopted in clinical practice. In recent years, however, fully automated methods for segmenting CT data have started to emerge (Besler et al., 2021; Bjornsson et al., 2023; Yosibash et al., 2020). Quantifying the accuracy, robustness, reproducibility, and repeatability of these segmentation tools is of major importance for research and potential down-stream translation of CT-based biomarkers into clinical practice. Here, we define.

• accuracy, as the ability of the method to predict ground truth segmentation;
• robustness, as the ability of the method to produce accurate results across varied cohorts (e.g. healthy vs. pathological) and across scanners;
• reproducibility, as the ability of the method to produce consistent results using the same CT dataset, thus pertaining to e.g. inter- and intra- operator variability;
• repeatability, as the ability of the method to produce the same results for the same subject and same scanner, in two separate imaging sessions.

It is important to highlight that besides fracture risk assessment, CT-based biomarkers may be valuable in quantifying the effects of treatments in a more detailed way than DXA-based approaches. For example, some clinical research studies have already demonstrated the ability of CT-based FE-derived biomarkers in monitoring treatment responses in individual patients and detecting changes that were missed by DXA (Keaveny et al., 2020). As such, fully automating the segmentation of CT images, to make the process fast and clinically applicable for image-based and FE-based biomarkers, is crucial for guiding personalized treatments.

The aim of this work was to systematically review the literature on clinical CT image segmentation methods for the bones in the human hip, to establish the current level of evidence to support the use of these methods for quantifying image-based bone biomarkers in large clinical cohorts. To this end we focused on the general conclusions that can be drawn from the literature on accuracy, robustness, reproducibility, and repeatability, and finally the availability of these segmentation methods for use in research and clinical practice.

## 2 Methods

### 2.1 Literature search

**FIGURE 1:**
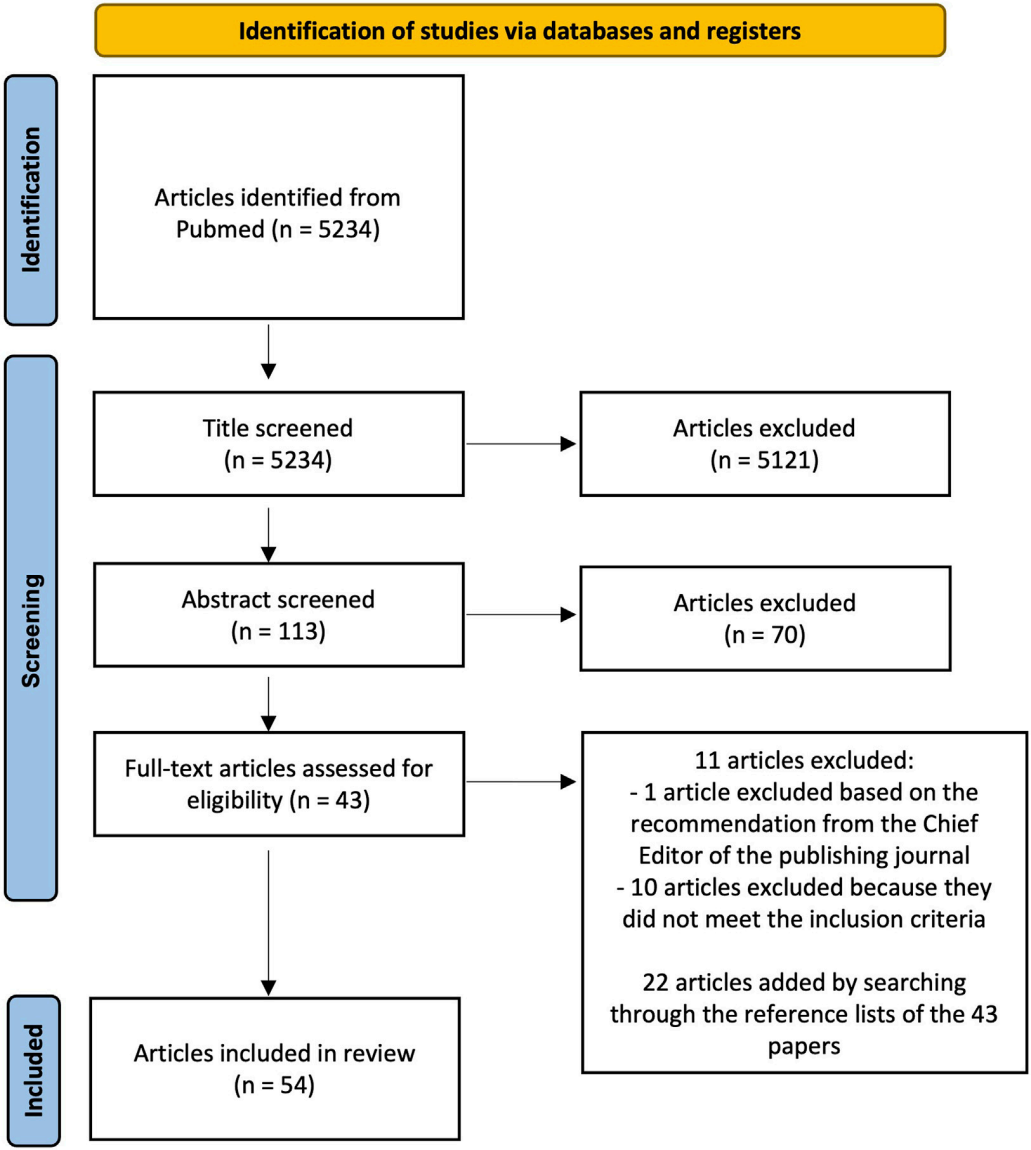
PRISMA flow chart of the systematic literature review

We conducted an electronic literature search on PubMed to identify relevant articles published until the end of July 2024. The following keywords were used as search terms: “image segmentation”, “femur segmentation”, “pelvis segmentation”, “automatic segmentation”, “convolutional neural network”, “fracture risk”, “computed-tomography”, “thresholding”, “statistical shape model”, “graph-cut”, “multi-atlas”, “deep-learning”, “bone strength”. The search terms were combined as follows: (((image segmentation) OR (femur segmentation) OR (pelvis segmentation)) AND ((automatic segmentation) OR (convolutional neural network) OR (thresholding) OR (statistical shape model) OR (graph-cut) OR (multi-atlas) OR (deep-learning)) AND ((computed-tomography) OR (fracture risk) OR (bone strength))). This resulted in 5234 articles. Using inclusion and exclusion criteria, authors CF and BH independently screened the studies based on title and subsequently, they compared their lists. Any disagreement between the two lists was resolved through discussion without in depth analysis of the content of the papers to reach a consensus leading to a total of 113 papers that were identified as being relevant for further review. These papers were subsequently screened based on their abstract by authors CF and BH independently, which further reduced the number of relevant articles to 43. During this second screening, any disagreement regarding the inclusion of papers was solved through a discussion. Finally, the full text of the 43 articles was evaluated to verify whether they met the inclusion criteria. This evaluation was performed independently by three different authors (CF, BH, VSC). In case of a disagreement, consensus on which articles to include was reached through discussion. If necessary, a fourth author (LME) was consulted to make the final decision. One paper among the 43 was excluded based on the recommendation from the Chief Editor of the publishing journal. Searching through the reference lists of these papers, additional publications of interest were found, resulting in a total of 54 papers being included in this review (Figure 1).

### 2.2 Inclusion and exclusion criteria

Studies that reported development of segmentation methods, validated on human femurs and/or pelvises were included in the review. Studies on other bones were excluded, as well as studies on fractured bones. Segmentation methods validated on both clinical and non-clinical CT data were included. Studies that used micro-CT images or data from other imaging modalities than clinical CT, were excluded. Only studies that reported a quantitative comparison between the study segmentation method and manual segmentation were included. Studies that did not report validation outcomes and/or resolution of images were excluded.

### 2.3 Comparing studies

All the papers included in this review were gathered into four tables, two for studies on the femur and two for studies on the pelvis. In terms of accuracy, four main metrics were extracted from the studies, i.e., DSC, JAC, HD, and its 95th percentile variant (HD95). All four metrics aim to quantify different aspects of the difference between ground truth and the segmented structures. The DSC measures the spatial overlap between the ground truth mask and the predicted mask and is given by the following equation (Dice, 1945):

$$DSC=\frac{2\left|GT\cap MS\right|}{\left|GT\right|+\left|MS\right|}=\frac{2TP}{2TP+FP+FNG}$$

where GT is the ground truth mask and MS the predicted mask (DSC=0, no overlap and DSC=1, full overlap). The JAC index represents a measure of similarity between two objects and is defined by the following equation:

$$JAC=\frac{\left|GT\cap MS\right|}{\left|GT\cup MS\right|}=\frac{TP}{TP+FP+FNG}$$

where GT is the ground truth and MS the predicted mask (JAC=0, the segmentations have no common member, JAC=1, the segmentations are identical). The HD and its 95% percentile variant represent the distance between the ground truth and the resulting segmentation. The Hausdorff distance is the maximum of all shortest distances for all points from one object’s boundary to the other. Assuming that A and B are two non-empty subsets, HD can be defined as follows:

$$HD=\max\left\{d\left(A,B\right),d\left(B,A\right)\right\}$$

with $d\left(A,B\right)=\max_{a\in A}\;\min_{b\in B}\;{\left\|a-b\right\|}_{2}$ and $d\left(B,A\right)=\max_{b\in B}\;\min_{a\in A}\;{\left\|a-b\right\|}_{2}$ . The 95th percentile variant of HD removes a small subset of outliers in $d\left(A,B\right)$ and $d\left(B,A\right)$ making the metric less sensitive to irregularities. In general, distance-based metrics, such as the HD, assess the accuracy of object boundaries and thus quantify outliers when segmentation masks are split into multiple objects, where they are supposed to be connected/closed together, which DSC and JAC are not able to quantify. Forest plots, generated in Matlab R2022a (MathWorks, MA, USA), were also used to summarize results across studies in terms of DSC.

DSC, JAC, and HD with HD95 have been chosen because they represent the state-of-the-art metrics when quantifying the accuracy of medical image segmentation methods, evaluated on ground truth data. However, not all studies have used these accuracy metrics. Thus, for those works, the accuracy measures used by the authors’ have been reported. In addition to the accuracy, based on the definitions reported in Section 1, robustness, reproducibility and repeatability outcomes of different studies were qualitatively compared.

### 2.4 Risk of bias

The risk of bias was evaluated in the following manner. First, for the four main topics investigated in this review (i.e., accuracy, robustness, reproducibility, and repeatability) authors CF and BH identified parameters that may affect the results reported in the studies. The type of CT dataset (i.e., if the dataset was obtained by scanning a homogeneous population or non-homogeneous population), and number of CT scanners used in the studies, were judged to be parameters that could bias the evaluation of the accuracy and robustness of the segmentation method. In terms of reproducibility, the risk of bias was evaluated to be associated with lack of quantification of inter- and intra-operator variability. Here, of importance are the number of operators involved in evaluating the inter-operator variability and the number of times each operator analyzed a CT dataset for the intra-operator variability. In terms of repeatability, the risk of bias was evaluated to be associated with absence of re-scanning of the same patient using the same scanner. An important aspect of the re-scanning procedure is the time between two imaging sessions, as changes in bone mass are time dependent due to e.g. use of pharmacological agents or simply due to aging. Subsequently, each study included in the review was independently evaluated by the two authors (CF and BH) based on the presence of these potential sources of bias. If a study reported a given parameter, it was labeled with a ‘Yes’, otherwise with a ‘No’. Articles that reported 0 or 1 parameters were classified as high risk of bias, whereas studies that reported 2 or 3 parameters were considered a source of medium risk of bias. If the articles reported all 4 parameters, they were assessed as providing low risk of bias.

## 3 Results

### 3.1 Search outcome

Supplementary Tables S1, S2 report the studies that validated segmentation methods on human femurs, whereas Tables 3, 4 include the studies validated on human pelvises. The studies are grouped in the tables based on the type of segmentation method under evaluation, i.e.: 1) threshold-based; 2) statistical shape method (SSM)-based; 3) atlas-based; 4) graph-cut based; and 5) convolutional neural network-based (CNN) methods (see Appendix A). In Supplementary Table S1 and Table 3 for each study the following information has been included: reference to the study, number of datasets segmented and type of material, scanning parameters and image resolution, metrics used for the assessment of accuracy, robustness, reproducibility, repeatability and remarks. The remarks column includes information that may be useful to the reader, such as the processing speed (when available), whether the method is fully automatic or semi-automatic, and whether the method is available under an open-source or a commercial license. In Supplementary Table S2; Table 4, the quantitative results for reported accuracy, robustness, reproducibility and repeatability have been reported for each study.

**TABLE 3:** Segmentation methods developed for the pelvis from the studies included in the review. The table shows the following information: reference of study; number of datasets N and type of material segmented; type of CT scanner, scanning parameters, and image resolution; segmentation method; metrics used to evaluate accuracy, robustness, reproducibility and repeatability; and remarks. NR: not reported

Study	N datasets segmented, type of material	CT-scanner, scanning parameters and resolution	Metrics used for accuracy, robustness, reproducibility and repeatability	Remarks
Threshold-based				
Zoroofi et al. (2003)	60 in-vivo CT datasets (120 hip joints) Among the 120 hip joints, THR had been performed on nine cases. Hence 111 hip joints were used for further evaluations Hip joints classified in 4 groups: 1) acetabulum and the femoral head are well separated from each other; 2) acetabulum and femoral head are close to each other; 3) acetabulum and femoral head are close to each other but the shape of the femoral head is different from that of a perfect ellipse, due to pathology and malformation of the pelvis and the femur; 4) acetabulum and femoral head are attached due to the severity of a bone disease	Device and scanning parameters NR 0.68x0.68x3 mm^3^ ⇒ segmentation algorithm performs a resampling to 0.68x0.68x0.75 mm^3^	Accuracy: ASD (mm), average DSC (%) Robustness: NR Reproducibility: NR Repeatability: NR	Automatic method Manual segmentation as the ground truth The developed method is not publicly available Average time: 7 min per hip; 9.5 s per slice
Anstey et al. (2011)	A formaldehyde-fixed cadaveric hemi-pelvis with all soft tissues intact	16-slice CT scanner (Lightspeed+ XCR, General Electric, Milwaukee, USA) Slice thickness of 0.625 mm	Accuracy: RMSE (mm), Average Deviation (unsigned, mm), Average Deviation (signed, mm), Max Deviation (unsigned, mm) Robustness: NR Reproducibility: NR Repeatability: NR	Semi-automatic method This study aimed to assess whether a plastic model of the hip joint can be accurately made from a pelvic CT scan. A cadaver hemi-pelvis was CT imaged and segmented from which a 3D plastic model of the proximal femur and hemi-pelvis were fabricated using rapid prototyping. Both the plastic model and the cadaver were then imaged using a high-resolution laser scanner. A three-way shape analysis was performed to compare the goodness-of-fit between the cadaver, image segmentation, and the plastic model. From laser scanning the STL were constructed (ground truth) The developed method is not publicly available
Zhou et al. (2013)	35 in-vivo CT datasets (70 hip joints) with a status ranging from healthy to severe osteoarthritis	GE Toshiba CT machine, field of view of 320 mm^2^ 0.73x0.73x1.5 mm^3^	Accuracy: JAC (%), RMSD (mm) Robustness: NR Reproducibility: NR Repeatability: NR	Automatic method The Authors developed a 3D adaptive thresholding method and compared the segmentation results with other common segmentation methods, such as global threshold method, level-set based method, FCM (fuzzy C-mens-based method) based method and Straka’s method (Straka et al., 2003) Manual segmentation as the ground truth The developed method is not publicly available Computation time: 12 min for each dataset, 8 s for a slice
Cheng et al. (2013)	110 in-vivo hips from patients that exhibited a wide range of bone pathology and morphometric variation	GE Toshiba CT machine 0.68x0.68x1.5 mm^3^	Accuracy: DSC (%), ASD (mm) Robustness: NR Reproducibility: NR Repeatability: NR	Automatic method Automatic approach for simultaneous segmentation of the femoral head and proximal acetabulum from 3D CT data. Based on several anatomical and imaging criteria,they classified the hips into four groups (G1,G2,G3,G4). Manual segmentation as the ground truth Comparison with (Zoroofi et al., 2003; Yokota et al., 2013) in terms of accuracy and execution time The developed method is not publicly available Computation time: - average time per slice: 9.5 s for (Zoroofi et al., 2003), 13.6 s for (Yokota et al., 2013), 8 s for proposed method - total time: 12.9 h for (Zoroofi et al., 2003), 18.5 h for (Yokota et al., 2013), 10.9 h for proposed method
SSM-based				
Lamecker et al. (2004)	23 in-vivo CT datasets	Device and scanning parameters NR 1.4x1.4x5 mm^3^	Accuracy: d_mean_ (mm), d_RMS_ (mm), HD (mm), d_r_ (%) Robustness: NR Reproducibility: NR Repeatability: NR	Semi–automatic method Manual segmentation as the ground truth The developed method is not publicly available
Seim et al. (2008)	50 in-vivo CT datasets	Device and scanning parameters NR 0.9x0.9x5mm^3^	Accuracy: AD (mm), ADRMS (mm), MD (mm) Robustness: NR Reproducibility: NR Repeatability: NR	Automatic method Manual segmentation as the ground truth The developed method is not publicly available Computation time: less than 5 minutes
Kainmueller et al. (2009)	50 in-vivo CT datasets of pelvis and 30 in-vivo CT datasets of femur	Device and scanning parameters NR Pelvis CT datasets: 0.9x0.9x5 mm^3^ Femur CT datasets: 0.5x0.5 mm^2^, slice distances of 0.5 to 1.5 mm	Accuracy: ASD (mm), DSC (%) Robustness: NR Reproducibility: NR Repeatability: NR	Automatic method The accuracy is evaluated on pelvis CT datasets Manual segmentation as the ground truth The developed method is not publicly available Computation time: 4 : 20 to 6 : 00 min
Audenaert et al. (2019)	250 in-vivo CT scans	Device and scanning parameters NR Pixel size between 0.575 mm to 0.975 mm	Accuracy: ADE (mm), MDE (mm) Robustness: NR Reproducibility: NR Repeatability: NR	Automatic method The segmented structures included the 6 lower vertebrae, sacrum, pelvis, femur, patella, fibula, tibia, talus, calcaneum, navicular, cuboid and three cuneiform bones. Accuracy was tested on 10 samples Manual segmentation as the ground truth The developed method is not publicly available Computation time: automatic segmentation of a full data set required on average 2 hours per case Fitting of the articulated SSM failed on three cases scanned with their legs crossed
Atlas-based				
Chu et al. (2015a)	30 in-vivo hip CT datasets (60 hip joints)	Device and scanning parameters NR Intra-slice resolutions of these 30 CT data ranged from 0.576 mm to 0.744 mm while the inter-slice resolutions were 1.6 mm for all CT data	Accuracy: ASD (mm), DSC (%) Robustness: NR Reproducibility: NR Repeatability: NR	Automatic method The Authors conducted a 15-fold cross validation study to evaluate the performance of their approach. The 30 CT data was randomly partitioned into 15 equal size subsets. Of the 15 subsets, each time a single subset (2 CT data) was used as the test data while the remaining 14 subsets were used as training data. This process was repeated 15 folds, with each one of the 15 subsets used exactly once as the test data. Manual segmentation as the ground truth The developed method is not publicly available Computation time: 3.1 min for segmentation of a hip joint
Chu et al. (2015b)	30 in-vivo hip CT datasets	Device and scanning parameters NR Intra-slice resolutions ranged from 0.576 mm to 0.744 mm while the inter-slice resolutions were characterized by a constant value of 1.6 mm	Accuracy: ASD (mm), DSC (%) Robustness: NR Reproducibility: NR Repeatability: NR	Automatic method FACTS (Fully Automatic CT Segmentation): combining fast random forest (RF) regression based landmark detection, multi-atlas-based segmentation, with articulated statistical shape model (aSSM) based fitting Same data of (Chu et al., 2015a) but different method. The method proposed in this work requires greater computation time and is less accurate with respect to (Chu et al., 2015a) Manual segmentation as the ground truth The developed method is not publicly available Computation time: 7.9 min per hip
Hanaoka et al. (2017)	50 in-vivo whole-torso CT datasets. All subjects had no bone diseases other than osteopenia.	Device and scanning parameters NR Voxel size: 0.977×0.977×1.250 mm	Accuracy: DSC (%), HD (mm), ADE (mm) Robustness: NR Reproducibility: NR Repeatability: NR	Automatic method Manual segmentation as the ground truth The developed method is not publicly available Computation time: 15 min for one segmentation task using 5 atlases, 110 min when 39 atlases were used
Convolutional neural network				
Wang et al. (2019)	90 in-vivo abdominal CT from two studies (50 from the CT colonography study, 40 from the lymph node study)	For 50 datasets from CT colonography: at least a 16 slice CT scanner, 0.5–1.0 mm collimation, pitch of 0.98– 1.5, matrix 512×512, field-of-view to fit, 50 effective mAs, 120 kVp, standard reconstruction algorithm, slice thicknesses of 1–1.25 mm with a 0.8 mm reconstruction interval. NR for lymph node study	Accuracy: DSC Robustness: NR Reproducibility: NR Repeatability: NR	Automatic method Manual segmentation as the ground truth The developed method is not publicly available For testing, the U-net prediction takes 20-30 seconds to process a 3D volume, and the shape model estimation takes 2-3 minutes for each pass
Noguchi et al. (2020)	32 in-vivo CT datasets i.e. 16 patients (for training and validation). Among the 16 patients, 9 patients had known sites of bone metastases. 20 in-vivo CT datasets (for testing robustness on other data sources) 27 in-vivo CT public datasets (20 patients)	For the 32 CT datasets: Aquilion 64, Aquilion ONE, Aquilion PRIME; Canon Medical Systems, Otawara, Japan; slice thickness was 0.5, 1.0, or 5.0 mm, and axial in-plane image resolution was 0.41–0.68 mm For the 20 CT datasets: Device and scanning parameters NR; slice thickness was 1 or 1.25 mm, and axial in-plane image resolution was 0.63–0.97 mm For the 27 CT public datasets: helical CT scanner (Philips, Amsterdam, The Netherlands); slice thickness of 5 mm and axial in-plane image resolution of 0.78 mm	Accuracy: DSC, JAC Robustness: it has been proved by considering three different datasets and testing three types of data augmentation techniques (conventional method, Mixup and RICAP) (DSC, JAC) Reproducibility: NR Repeatability: NR	Automatic method To compare the proposed model with those of previous studies, the network was trained and validated on a publicly available labelled dataset (27 CT datasets). Of the 27 examinations, 15 were used for training, 3 for validation, and 9 for testing. Manual segmentation as the ground truth The developed method is not publicly available The training time was ∼2 h per fold for the 32 CT datasets. Training time was approximately 40 min per split for the 27 CT public datasets
González Sánchez et al. (2020)	30 in-vivo dual energy CT	Siemens SOMATOM, low energy (mostly 80 kV), high energy (mostly 150 kV), mixed images (around 120 kV) Isotropic voxel size ranged 0.67x0.67x1 mm^3^ to 0.977x0.977x1.0 mm^3^	Accuracy: DSC Robustness: NR Reproducibility: NR Repeatability: NR	Automatic method Manual segmentation as the ground truth The developed method is publicly available Computation time: 5 s
Hiasa et al. (2020)	20 in-vivo CT volumes scanned (Osaka University Hospital THA dataset)	Device and scanning parameters NR Field of view 360 × 360 mm^2^, matrix size 512 × 512 Slice intervals: 2.0 mm for the region including the pelvis and proximal femur, 6.0 mm for the femoral shaft region, and 1.0 mm for the distal femur region	Accuracy: DSC (%), AD (mm) Robustness: NR Reproducibility: NR Repeatability: NR	Automatic method The Osaka University Hospital THA dataset was used for training and cross-validation for the accuracy evaluation and prediction of the DSC coefficient Manual segmentation as the ground truth The developed method is not publicly available Average training time: 11 hours Average computation time for the inference on one CT volume with about 500 2D slices was approximately 2 minutes excluding file loading, and the post-processing took about 3 minutes
Jeuthe et al. (2021)	8 in-vivo CT datasets for development and 30 in-vivo CT datasets for testing	8 datasets at 120 kV, different scanners, voxel size ranged from 0.7x0.7x1.0 mm^3^ to 0.9x0.9x3.0 mm^3^ 30 datasets from Siemens SOMATOM Force scanner, 80 kV, 150 kV, voxel size ranged from 0.63x0.63x1.0 mm^3^ to 0.98x0.98x1.0 mm^3^	Accuracy: DSC Robustness: NR Reproducibility: NR Repeatability: NR	Automatic method MK2014v2, JS2016 and JS2018 algorithms Manual segmentation as the ground truth The developed method is not publicly available
Liu et al. (2021)	1184 in-vivo 3D volumes (entire dataset) from 7 CT sub-datasets (ABDOMEN 35, COLONOG 731, MSD_T10 155, KITS19 44, CERVIX 41, CLLINIC 103, CLINIC-metal 75)	Device and scanning parameters NR Mean spacing entire CT dataset: 0.78x0.78x1.46 (mm)	Accuracy: DSC, HD (mm) Robustness: Six deep networks have been trained, one network per single sub-dataset and tested on each sub-dataset: DSC, HD (mm) Reproducibility: NR Repeatability: NR	Automatic method Manual segmentation as the ground truth The developed method is publicly available
Xu et al. (2022)	35 in-vivo CT scans from the Cancer Imaging Archive	Device and scanning parameters NR (0.78±0.11) × (0.77±0.1) × (0.96±0.17) mm^3^	Accuracy: DSC (%), GapDSC (%), HD (#voxels) Robustness: NR Reproducibility: NR Repeatability: NR	Automatic method Use of 2D image slices from different views helped to produce accurate multi-segmentation despite the small dataset. Post-processing step corrects for misclassification near midline (e.g. left or right pubis) Pretraining (inferior segmentation) =2 Fine tuning (uses accurate segmentation) =2 Initial predict then manual correct, then repeat fine tuning process=2 Evaluation cases=21 Manual segmentation as the ground truth The developed method is not publicly available
Wu et al. (2022)	815 in-vivo CT datasets from 5 sub-datasets: normal hip dataset, osteoarthritis (OA) hip-joint dataset, dysplasic hip (DDH) dataset, femoral neck fracture (FNF) hip joint dataset, osteonecrosis of femoral head (ONFH) hip joint dataset	Scanning parameter NR Phillip CT Brilliance ICT with 1.00-mm slice thickness and 512×512 image resolution	Accuracy: DSC, HD (mm) Robustness: evaluated using diseased hip datasets (DSC, HD (mm)) Reproducibility: NR Repeatability: NR	Automatic method Manual segmentation as the ground truth Computation time: 23.7±1.0 s on a Nvidia GeForce GTX TITAN X GPU The developed method is not publicly available
Zhai et al. (2023)	81 in-vivo CT images (31 preoperative images of diseased hips, and 50 healthy hip images). Hip disorders of the 31 cases included osteonecrosis of femoral head, osteoarthritis, developmental dysplasia of the hip, femoral neck fracture, and bone tumors.	31 CT scans acquired with the Somatom Definition Flash scanner (Siemens Medical Solutions, Erlangen, Germany), 120 kVp, 336 mA, 1 mm slice thickness, 512 × 512 matrix size, 0.62–0.98 mm pixel spacing 50 CT scans acquired with multidetector row CT scanners, 120 kVp, 1–1.25 mm slice thickness, 512 × 512 matrix size, 0.60–0.98 mm pixel spacing	Accuracy: DSC, HD95 (mm) Robustness: NR Reproducibility: NR Repeatability: NR	Automatic method Manual segmentation as the ground truth Computation time: 10 s The developed method is not publicly available
Other methods				
Guo et al. (2018)	50 in-vivo hip CT datasets	Hip joints were acquired on a Philips Brilliance 64 CT scanner 0.68x0.68x0.67 mm^3^	Accuracy: evaluated on 10 hip joints for the three different segmentation methods (ASD (mm), DSC (%), TPR (%)) Robustness: NR Reproducibility: NR Repeatability: NR	Automatic method Bone segmentation framework based on a consideration of the surface normal direction A comparison with two recently published methods (Yokota’s and Chandra’s methods) (Chandra et al., 2014; Yokota et al., 2013) was performed. Yokota’s and Chandra’s methods need training data, so fivefold cross-validations were performed for Yokota’s and Chandra’s methods. In the fivefold cross-validation, 50 hip joints were randomly divided into five groups with the same size (each group has 10 hip joints), then four groups (40 hip joints) were used for training and the remaining one (10 hip joints) for testing. This operation was repeated five times, each time three methods used the same group as testing data, and the average is the final result. A comparison to Yao’s method (Yao et al., 2005) was performed. Proposed method and Yao’s algorithm are an unsupervised approach, they do not need any training data; thus, fivefold cross-validations were not used for this comparison. Manual segmentation as the ground truth The developed method is not publicly available

**TABLE 4:** Segmentation methods developed for the pelvis from the studies included in the review. The table shows the quantitative results obtained from each study for evaluating accuracy, robustness, reproducibility and repeatability. NR: not reported.

Study	Accuracy	Robustness	Reproducibility	Repeatability
Threshold-based				
Zoroofi et al. (2003)	ASD 0.91 mm, average DSC (%) 93.89	NR	NR	NR
Anstey et al. (2011)	RMSE (mm): cadaver to segmentation 0.61, model to segmentation 0.49, cadaver to model 0.48. Average Deviation (unsigned, mm): cadaver to segmentation 0.58, model to segmentation 0.47, cadaver to model 0.42 Average Deviation (signed, mm): cadaver to segmentation -0.49, model to segmentation -0.46, cadaver to model -0.32 Max Deviation (unsigned, mm): cadaver to segmentation 1.62, model to segmentation 0.94, cadaver to model 1.58 mm	NR	NR	NR
Zhou et al. (2013)	JAC: 79.8% (range 74.4–83.0%) by global threshold method, 85.6% (range 81.2–89.2%) by FCM method, 89.1% (range 86.0–91.6%) by Straka’s method (Straka et al., 2003), 95.2% (range 93.4–96.9%) by level set method, and 96.4% (range 95.1–97.6%) by the proposed method RMSD (mm): 0.75 (range 0.59–0.88) by global threshold method, 0.64 (range 0.49–0.85) by FCM method, 0.56 (range 0.43–0.71) by Straka’s method (Straka et al., 2003), 0.45 (range 0.32–0.68) by level set method, and 0.38 mm (range 0.25–0.53) by the proposed method	NR	NR	NR
Cheng et al. (2013)	DSC (%): G1: 94.53 using method in (Zoroofi et al., 2003), 92.85 using method in (Yokota et al., 2013), 95.09 proposed method; G2: 93.25 using method in (Zoroofi et al., 2003), 92.53 using method in (Yokota et al., 2013), 93.78 proposed method; G3: 89.92 using method in (Zoroofi et al., 2003), 89.04 using method in (Yokota et al., 2013), 91.01 proposed method; G4: 80.57 using method in (Zoroofi et al., 2003), 87.86 using method in (Yokota et al., 2013), 81.83 proposed method Average DSC (%): 90.36 using method in (Zoroofi et al., 2003), 90.14 using method in (Yokota et al., 2013), 91.55 proposed method Standard deviation DSC (%): 5.31 using method in (Zoroofi et al., 2003), 1.95 using method in (Yokota et al., 2013), 4.82 proposed method ASD (mm): G1: 0.70 using method in (Zoroofi et al., 2003), 0.86 using method in (Yokota et al., 2013), 0.65 proposed method; G2: 1.12 using method in (Zoroofi et al., 2003), 1.33 using method in (Yokota et al., 2013), 1.07 proposed method; G3: 1.34 using method in (Zoroofi et al., 2003), 1.71 using method in (Yokota et al., 2013), 1.25 proposed method; G4: 2.49 using method in (Zoroofi et al., 2003), 1.80 using method in (Yokota et al., 2013), 2.26 proposed method Average ASD (mm): 1.31 using method in (Zoroofi et al., 2003), 1.49 using method in (Yokota et al., 2013), 1.22 proposed method Standard deviation ASD (mm): 1.12 using method in (Zoroofi et al., 2003), 1.04 using method in (Yokota et al., 2013), 0.98 proposed method	NR	NR	NR
SSM-based				
Lamecker et al. (2004)	d_mean_ (mm): 0.6±0.2 d_RMS_ (mm): 0.8±0.3 HD (mm): 4.7±1.6 d_r_ (%): 1.3±1.6	NR	NR	NR
Seim et al. (2008)	For the complete pelvis: AD of 0.7±0.3 mm, AD_RMS_ of 1.9±0.6 mm, MD of 16.5±5 mm. Right hip bone: AD 0.4±0.1 mm, AD_RMS_ 1.1±0.3 mm, MD 9.2±2 mm. Left hip bone: AD 0.6±0.2 mm, AD_RMS_ 1.5±0.3 mm, MD 10.8±2.4 mm.	NR	NR	NR
Kainmueller et al. (2009)	ASD (mm): 0.30 for right hip bone, 0.60 for left hip bone. DSC (%): 94.90 for right hip bone and 92.01 for left hip bone	NR	NR	NR
Audenaert et al. (2019)	ADE (mm): pelvis 0.75±0.17 femur 0.65±0.10 MDE (mm): pelvis 7.84±2.26 femur 4.79±2.39	NR	NR	NR
Atlas-based				
Chu et al. (2015a)	ASD (mm): 0.30 for pelvis and femur DSC (%): 97.80 for pelvis and femur	NR	NR	NR
Chu et al. (2015b)	ASD (mm): 0.37 for pelvis and femur DSC (%): 96.80 for pelvis and both femurs	NR	NR	NR
Hanaoka et al. (2017)	DSC (%): 90±2 (using 5 atlases) HD (mm): 5.30±2.14 (using 5 atlases) ADE (mm): 0.59±0.14 (using 5 atlases)	NR	NR	NR
Convolutional neural network				
Wang et al. (2019)	DSC: left femur 0.958±0.031; right femur 0.962±0.018; left hip 0.958±0.013; right hip 0.957±0.011; sacrum 0.924±0.027	NR	NR	NR
Noguchi et al. (2020)	32 CT datasets: best DSC: 0.983±0.005 best JAC: 0.968±0.009 20 CT datasets: best DSC: 0.943±0.007 best JAC: 0.898±0.010 27 CT datasets: best DSC: 0.947±0.013 best JAC: 0.899±0.023	32 CT datasets: - DSC 0.981 ± 0.004, JAC 0.962 ± 0.008 using conventional method - DSC 0.981 ± 0.005, JAC 0.963 ± 0.009 using Mixup method - DSC 0.983 ± 0.005, JAC 0.967 ± 0.010 using RICAP method 20 CT datasets: - DSC 0.947 ± 0.010, JAC 0.904 ± 0.015 using conventional method - DSC 0.906 ± 0.045, JAC 0.846 ± 0.058 using Mixup method - DSC 0.946 ± 0.008, JAC 0.902 ± 0.012 using RICAP method 27 CT datasets: - DSC 0.942 ± 0.014, JAC 0.892 ± 0.025 using conventional method - DSC 0.892 ± 0.037, JAC 0.809 ± 0.058 using Mixup method - DSC 0.943 ± 0.014, JAC 0.893 ± 0.024 using RICAP method	NR	NR
González Sánchez et al. (2020)	DSC: 0.976	NR	NR	NR
Hiasa et al. (2020)	DSC (%): 98.1±0.43 AD (mm): 0.145±0.040	NR	NR	NR
Jeuthe et al. (2021)	DSC: 0.914 (mean value) with a min of 0.714	NR	NR	NR
Liu et al. (2021)	3D U-Net cascade with the deep network model trained on entire dataset: - left hip DSC=0.989 and HD=4.24 mm - right hip DSC=0.991 and HD=3.03 mm	Six deep networks have been trained, one network per single sub-dataset and tested on each sub-dataset: - best average DSC=0.989 - best average HD=1.93 mm	NR	NR
Xu et al. (2022)	DSC=98.63±0.56 GapDSC=96.47±1.60 HD (#voxels) =3.67±1.13	NR	NR	NR
Wu et al. (2022)	Normal hip dataset: - mean DSC=0.9899 - mean HD=5.26 ± 0.6 mm	Diseased hip datasets: DSC=0.9355±0.0557 HD=4.19±1.04 mm	NR	NR
Zhai et al. (2023)	Left hip: - DSC=0.9737±0.0075, HD95=2.03±0.14 (mm) Right hip: - DSC=0.9713±0.0170, HD95=2.07±0.26 (mm)	NR	NR	NR
Other methods				
Guo et al. (2018)	Accuracy on 10 hip joints for the three different segmentation methods ASD (mm): - pelvis: Yokota’s 0.55±0.15, Chandra’s 0.51±0.12, proposed method 0.42±0.08 - left femoral head: Yokota’s 0.51±0.12, Chandra’s 0.46±0.10, proposed method 0.38±0.07 - right femoral head: Yokota’s 0.52±0.11, Chandra’s 0.47±0.12, proposed method 0.39±0.08 DSC (%): - pelvis: Yokota’s 95.82±1.55, Chandra’s 96.47±1.42, proposed method 97.34±0.56 - left femoral head: Yokota’s 96.73±1.17, Chandra’s 97.34±1.26, proposed method 98.06±0.58 - right femoral head: Yokota’s 96.26±1.12, Chandra’s 96.91±1.08, proposed method 97.73±0.47 TPR (%): - pelvis: Yokota’s 93.35±2.43, Chandra’s 93.98±3.02, proposed method 95.86±1.48 - left femoral head: Yokota’s 93.46±3.30, Chandra’s 94.80±2.92, proposed method 96.34±1.27 - right femoral head: Yokota’s 93.92±2.86, Chandra’s 95.37±4.12, proposed method 96.83±1.22 Comparison of Yao’s method with proposed methos on 50 hip joints ASD (mm): - pelvis: proposed method 0.42±0.09, Yao’s 0.46±0.12 - left femoral head: proposed method 0.38±0.05, Yao’s 0.42±0.06 - right femoral head: proposed method 0.39±0.08, Yao’s 0.41±0.09 DSC (%): - pelvis: proposed method 97.32±0.52, Yao’s 95.71±0.71 - left femoral head: proposed method 98.03±0.53, Yao’s 96.61±0.72 - right femoral head: proposed method 97.72±0.33, Yao’s 96.68±0.62 TPR (%): - pelvis: proposed method 96.12±1.67, Yao’s 94.65±1.94 - left femoral head: proposed method 96.77±1.82, Yao’s 95.05±2.24 - right femoral head: proposed method 96.68±1.53, Yao’s 94.95±2.33	NR	NR	NR

### 3.2 Accuracy

**FIGURE 2:**
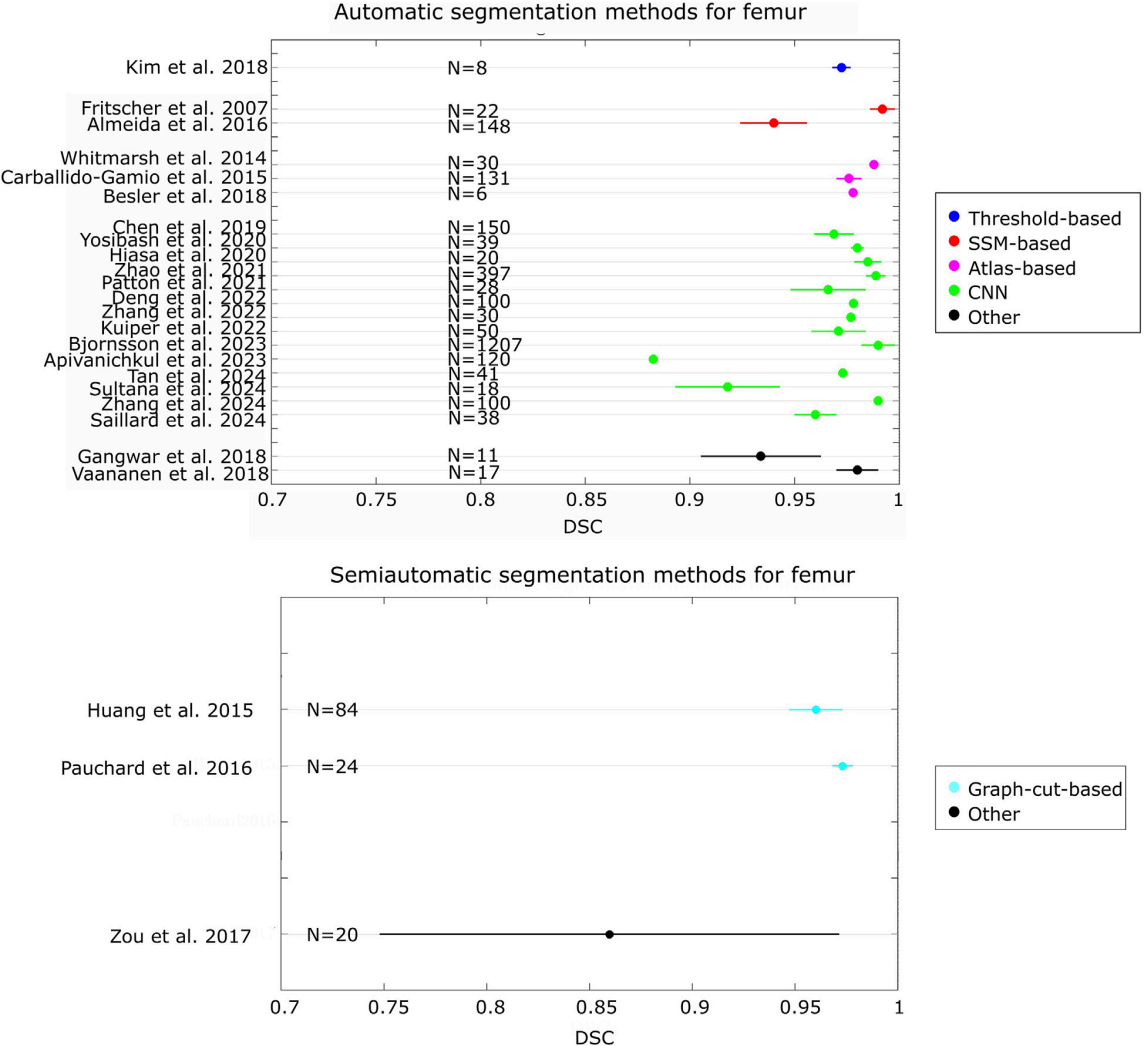
Forest plot that presents the level of DSC achieved by the automatic and semiautomatic segmentation methods developed for the femur. For each study, the number (N) of samples has been reported

**FIGURE 3:**
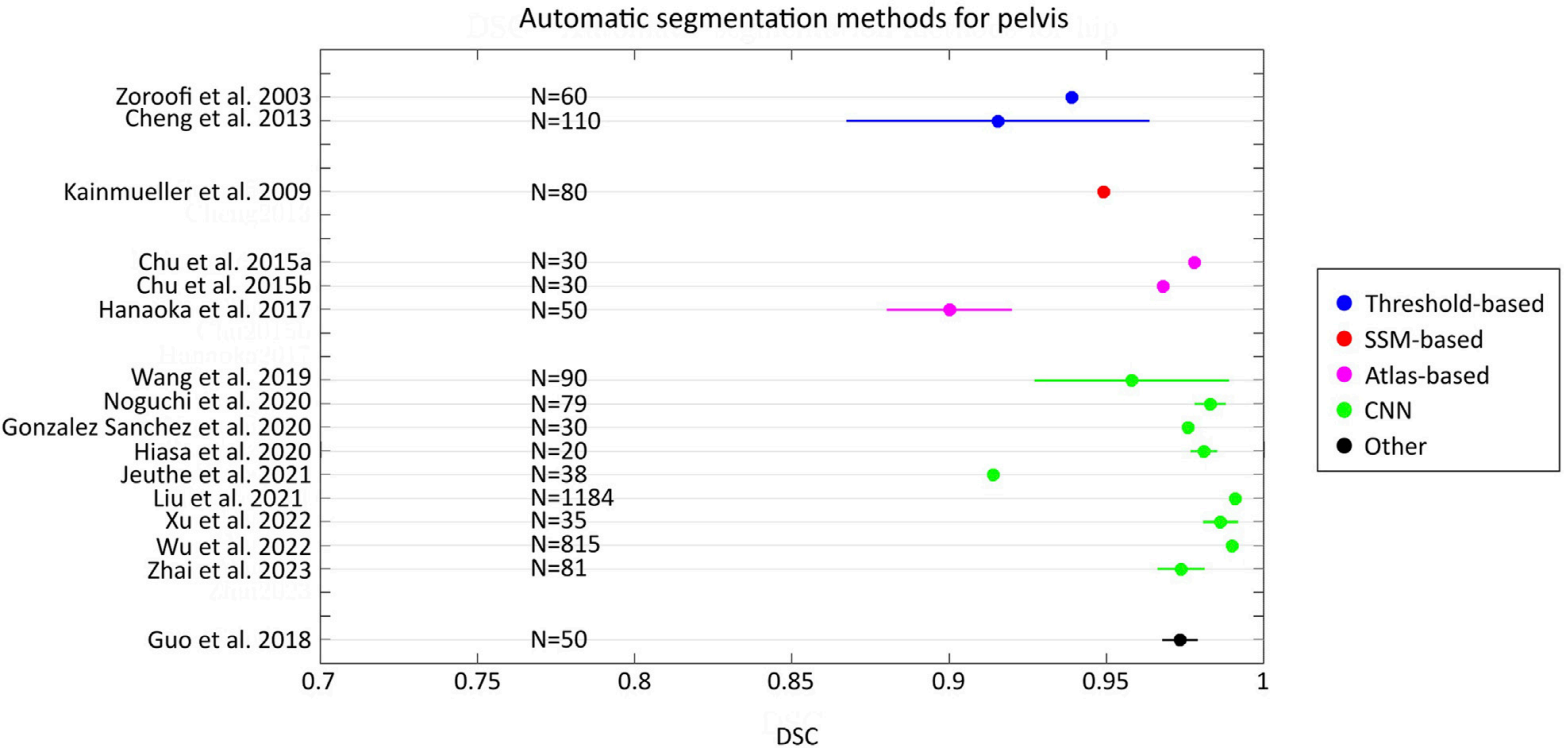
Forest plot that presents the level of DSC achieved by the automatic segmentation methods developed for the pelvis. For each study, the number (N) of samples has been reported

By comparing the state-of-the-art accuracy metrics used in medical image segmentation (i.e., DSC, JAC and HD/HD95), it can be observed that no studies evaluated the accuracy using all these metrics. In addition, in some studies, these metrics have not been used but other metrics quantified instead. More specifically, some studies used different distance-based metrics from HD, such as the average surface distance, the root mean-square average symmetric distance, the average distance error, and adopted volumetric-based metrics (e.g. volumetric overlap global error, volume difference). Furthermore, although HD95 represents a metric widely used in the field of image segmentation for its ability to handle outliers (Chen et al., 2021; Fick et al., 2021; Li et al., 2022; Van Den Oever et al., 2022), its use is not common for femur/pelvis segmentation. Only three studies reported this metric (Bjornsson et al., 2023; Kuiper et al., 2022; Zhai et al., 2023). Figures 2, 3 show the level of accuracy reached by the segmentation methods for the femur (Figure 2) and the pelvis (Figure 3) in terms of DSC. Only the studies that have used these metrics have been included when generating these figures.

### 3.3 Robustness

Only a few of the studies attempted to quantify robustness. In the study performed by Kang et al. (2003) on human femurs, the robustness of the segmentation method was assessed with respect to the image noise. Gaussian noise was artificially added to the CT data of the European Spine Phantom (ESP) that was used to calibrate the CT data. The authors showed that an increase in noise caused an increase in the segmented periosteal volume. Moreover, they showed that the segmented endosteal volume was initially constant at low noise but then increased with increasing noise. They also showed that the effect of noise increase was more pronounced when the cortical thickness was smaller. The authors also added noise to a CT dataset of femurs and showed that only in the noisiest image a sub-optimal segmentation was obtained, and user interaction became necessary. To test the robustness of the segmentation method, Fritscher et al. (2007) added image artifacts that imitated screws inside the femur and a black blob. They concluded that the segmentation of this dataset resulted in closely similar segmentation results for the same dataset without artifacts, demonstrating the robustness of the method. However, they did not report a quantitative evaluation of robustness. Carballido-Gamio et al. (2015) demonstrated the robustness of their atlas-based method using 80 scans of older women from two different clinical sites and two highly anisotropic spatial resolutions and reported a mean DSC of 0.976, a mean SYM of 0.203 mm, and a mean modified HD of 0.253 mm (HD=3.928 mm). Almeida et al. (2016) reported the robustness of their developed SSM-based method in terms of the percentage of successfully completed segmentations. They obtained a success rate of 98% from 148 datasets; only 3 (i.e. 2% of datasets) failed to converge, despite the significant variability in gender and age-related bone loss. Väänänen et al. (2019) tested their automatic segmentation method on in-vivo and ex-vivo CT datasets, which were acquired using different CT scanners and with different scanning parameters, thus corroborating the robustness of the method against changes in imaging parameters. They reported the robustness results in terms of DSC obtaining values of 0.93±0.02 and 0.98±0.01 for the in-vivo and ex-vivo CT datasets, respectively. In the study performed by Noguchi et al. (2020), the robustness of their CNN-based automatic segmentation was demonstrated by considering three different datasets, i.e. in-house dataset (32 CT datasets), secondary dataset (20 CT datasets) and public dataset (27 CT datasets), and testing three types of data augmentation techniques (conventional method, Mixup and RICAP). For the in-house dataset they reported a DSC of 0.983±0.005 and a JAC index of 0.968±0.009 without using data augmentation, with none of the three data augmentation techniques improving the results. On the secondary dataset, their method achieved a DSC of 0.943±0.007 and a JAC index of 0.898±0.010. In this case, conventional augmentation and RICAP improved the prediction accuracy (small improvements with the DSC increase of 0.002–0.004). In contrast, Mixup worsened the results. On the public dataset, using a combination of conventional augmentation and RICAP, a DSC of 0.947±0.013 and a JAC index of 0.899±0.023 were obtained. The robustness of the method developed by Liu et al. (2021) was assessed by using a large dataset of pelvic CT images pooled from multiple sources with which the authors performed a series of experiments. First, they tested the segmentation approach using all CT images together from all sources. Subsequently, they trained six deep networks, one network per single sub-dataset, and then tested them on each sub-dataset. Zhao et al. (2021) did not report any robustness quantification of their deep learning-based approach but they stated that the robustness of the method had been guaranteed by performing the data augmentation since the sample size was insufficient to train a precise 3D segmentation model. Data augmentation used to improve the robustness of the method has been also used by Deng et al. (2022). However, according to the authors’ opinion, using only data augmentation was not enough to guarantee the robustness of the method. It was crucial to perform additional tests, such as evaluating the method on a different dataset or cohort. Wu et al. (2022) validated their segmentation method using different CT datasets that included normal and pathological hip joints. They obtained DSC values of 0.9899±0.0014 and 0.9355±0.0557 on normal and diseased hip CT datasets, respectively. Bjornsson et al. (2023) evaluated the robustness of their method by involving two different samples of the AGES data set (a first sample characterized by 48 gold standard manually delineated proximal femur segmentations from 24 CT images and a second sample characterized by 1207 manually delineated segmentations, generated with a semi-automated delineation protocol). They quantified the robustness in terms of the DSC and HD95 reporting a mean DSC of 0.990±0.008 and a mean value of HD95 of 0.999±0.331 mm. Kuiper et al. (2022) proposed a deep-learning-based approach for automatic segmentation of bones. They used 50 CT datasets for both training and initial evaluation of the networks. Then, to evaluate the robustness of the method, they selected 10 CT datasets taken from a different image database with respect to the database from which the 50 CT datasets have been selected, in which the CT images have been acquired with different acquisition parameters and characterized by different subject demographics. The robustness was quantified in terms of mean absolute surface distance (MASD) and HD. For femurs, they reported values of 0.58±0.07 mm and 5.03±3.20 mm for MASD and HD, respectively.

### 3.4 Reproducibility

Four studies investigated reproducibility. Testi et al. (2001) evaluated the reproducibility using an in-vivo femur CT dataset and by extracting from the dataset 19 images uniformly distributed along the scanning plan. For every slice, both the endosteal and periosteal surfaces were extracted using both the border-tracing method developed by the authors and a threshold-based method. The geometry extraction was executed three times for both methods. The distance between the contours was reported in terms of HD. Three sets of contours for both extraction procedures were obtained. The comparison among the contours were reported in terms of RMSE of the HD for all slices. They showed that the border-tracing algorithm improved the reproducibility by about 40% with respect to the threshold-based method with a mean RMSE that decreased from 2.29 mm to 1.41 mm. Furthermore, Testi et al. (2001) performed a reproducibility study by using 6 CT data sets of patients in need of a custom-made prosthesis (CMP), considering only the first five proximal slices from every dataset. All images had been traced three times by both border-tracing and threshold-based methods. The inner and outer contours were compared to each other in terms of HD. The border-tracing method improved the HD from 5 to 1.5 mm with respect to the threshold-based procedure. Kang et al. (2003) performed an inter- and intra-operator study analyzing the datasets of 9 patients and evaluating the CV_RMS_ of the bone volume and cortical thickness within a sphere centered at the femoral neck. For the inter-operator variability, three operators analyzed each data set once, blinded to the results of the other operators. For the intra-operator variability, one operator analyzed each dataset three times. For inter- and intra-operator variability, the CV_RMS_ was always lower than 1% for trabecular and total bone volumes (0.27% ± 0.15% and 0.73% ± 0.43%, respectively, for inter-operator variability; 0.29% ± 0.17% and 0.64% ± 0.37%, respectively, for intra-operator variability) and below 2% for cortical thickness (1.71% ± 1.10% for inter-operator variability; 1.54% ± 1.10% for intra-operator variability). No significant differences were detected for inter- and intra-operator analyses. Pauchard et al. (2016) performed an inter-operator reproducibility study on 12 femurs involving three operators. Average mean surface-to-surface distance, DSC and HD were calculated between graph cut segmentations and manual segmentations from three operators in pair-wise manner. Pair-wise comparison of segmentation methods between operators indicated that HD measurements were consistently smaller between manual segmentations (maximum value 3.09 mm for manual segmentations vs. 3.49 mm for graph-cut segmentations). In contrast, mean surface-to-surface differences were consistently smaller, and DSC was higher between graph cut segmentations (maximum value of mean surface-to-surface distance 0.378 mm for manual segmentations vs. 0.006 mm for graph-cut segmentations; maximum value of DSC coefficient 0.980 for manual segmentations vs. 0.995 for graph-cut segmentations). Besler et al. (2021) evaluated the inter-operator reproducibility (three operators) measuring the SD_RMS_ (absolute units) and CV_RMS_ (%) for volume, integral density, and failure load in both cadaveric and in-vivo CT images, showing that their proposed segmentation algorithm considerably improved inter-operator reproducibility for all three outcomes (SD_RMS_ (CV_RMS_) equal to 9.58 mL (5.41%), 2.02 mg/cc (0.65%), and 70.10 N (5.17%) for volume, density, and failure load, respectively, using in-vivo CT datasets, and equal to 7.26 mL (4.10%), 1.86 mg/cc (0.92%), and 34.10 N (6.43%) in cadaveric CT datasets).

### 3.5 Repeatability

We found only two studies that investigated the repeatability of the segmentation method, in terms of its ability to produce the same results on the same subject and the same scanner, from two separate imaging sessions (Carballido-Gamio et al., 2015; Zhang et al., 2024). In that study (Carballido-Gamio et al., 2015), repeatability was evaluated using repeated scans after repositioning, on 22 subjects obtained on CT imaging systems from two manufacturers. Reproducibility was assessed with CV_RMS_ for ten compartmental vBMD parameters, seven compartmental tissue volume parameters, FE-derived bone strength under two loading conditions, three compartmental surface-based cortical bone thickness parameters, and three compartmental surface-based cortical vBMD parameters. Repeatability was also assessed in a local manner for three surface-based cortical bone thicknesses and three surface-based cortical vBMD parameters. In the study by Zhang et al. (Zhang et al., 2024) repeatability was assessed using 5 subjects, by repeating CT scans after repositioning and quantifying regional vBMD measures. The study showed a nominal value of around -0.01% for the mean difference between vBMD estimates from baseline and repeated scans. Moreover, the authors reported an intraclass correlation coefficient (ICC) between vBMD values measured from baseline scans and those obtained from repeated scans equal to 0.996, and a root-mean-square coefficient of variation of 0.72%.

### 3.6 Additional remarks

We made several additional observations based on Supplementary Tables S1, S2 and Tables 3, 4. First, only a few of the studies reviewed provided information about total operator time required to process each image (see Supplementary Tables S1 and Table 3). However, this information is essential for translation into clinical practice. Second, the segmentation methods have in some of the studies been evaluated on a downstream FE modelling workflow, to assess the ability of these methods in predicting femoral strength and strains. Väänänen et al. (2019) showed a good agreement between FE predicted strains derived from their automatic segmentation method using the Stradwin segmentation tool, and the corresponding ex-vivo measurements (R^2^=0.89, maximum error=27%, normalized RMSE=6%). A graph-cut segmentation (Pauchard et al., 2016) and CNN segmentation (Bjornsson et al., 2023) resulted in high correlation between FE predicted femoral strength compared to FE predicted strength derived from manual segmentation (R^2^=0.98 in (Pauchard et al., 2016); R^2^=0.988, RMSE=212.2 N, max difference=25.3% for left femurs and R^2^=0.986, RMSE=177 N, max difference=30.1% for right femurs in Bjornsson et al. (2023)). These results demonstrate the potential of these methods in producing reliable segmentations that can be used in an FE workflow for fracture risk assessment. The potential of automatic segmentation methods for FE-based femur fracture risk assessment was also demonstrated by Kim et al. (2018) that showed that their automatic segmentation method based on the complementary characteristics between the thresholding method and watershed algorithm was able to obtain a fracture risk prediction close to that obtained by the manual segmentation with an average relative error of 4.99%. Finally, the studies included in this review used different CT scanners, CT scanning parameters and resolutions. It is challenging to determine how these variables affect accuracy and robustness, as the scanner and scanning parameters are rarely used as isolated study variables. Similar conclusions can be drawn for the reproducibility and repeatability, which we found only sparsely addressed in the literature.

### 3.7 Risk of bias

Table 5 lists the summary of the risk of bias assessment. As can be observed, most of the studies presented a high risk of bias, whereas a small portion of the works demonstrated a medium risk. Most studies scored poorly on inter- and/or intra-operator variability and re-scanning of the same patient using the same CT scanner. These results can be explained by the fact that the studies included in the review did not quantify the accuracy, robustness, reproducibility, and repeatability of the segmentation method at the same time, but they focused on one or two of these metrics.

**TABLE 5:** Risk of bias assessment summary. The studies are reported in the same order as in Supplementary Tables S1, S2; Tables 3, 4.

Study	Parameters to evaluate the risk of bias				Risk of bias
	Heterogeneous CT dataset	Uses more than one CT scanner	Inter- and/or intra-operator variability study	Re-scanning the same patient using the same CT scanner	
Femur					
Kim et al. (2018)	No	No	No	No	High
Fritscher et al. (2007)	No	No	No	No	High
Zhang et al. (2014)	No	No	No	No	High
Almeida et al. (2016)	Yes	Yes	No	No	Medium
Whitmarsh et al. (2014)	No	No	No	No	High
Carballido-Gamio et al. (2015)	Yes	Yes	No	Yes	Medium
Besler et al. (2018)	No	No	No	No	High
Krcah et al. (2011)	No	No	No	No	High
Huang et al. (2015)	No	No	No	No	High
Pauchard et al. (2016)	Yes	No	Yes (inter-operator variability study, 3 operators)	No	Medium
Besler et al. (2021)	Yes	No	Yes (inter-operator variability study, 3 operators)	No	Medium
Aldieri et al. (2024)	No	No	No	No	High
Chen et al. (2019)	No	No	No	No	High
Yosibash et al. (2020)	Yes	Yes	No	No	Medium
Hiasa et al. (2020)	No	No	No	No	High
Zhao et al. (2021)	Yes	No	No	No	High
Patton et al. (2021)	Yes	No	No	No	High
Deng et al. (2022)	No	No	No	No	High
Zhang et al. (2022)	No	No	No	No	High
Kuiper et al. (2022)	Yes	Yes	No	No	Medium
Bjornsson et al. (2023)	Yes	No	No	No	High
Apivanichkul et al. (2023)	Yes	No	No	No	High
Tan et al. (2024)	No	No	No	No	High
Sultana et al. (2024)	No	No	No	No	High
Zhang et al. (2024)	No	No	No	Yes	High
Saillard et al. (2024)	Yes	Yes	Yes	No	Medium
Testi et al. (2001)	Yes	No	No	No	High
Kang et al. (2003)	No	No	Yes (inter- and intra-operator variability study, 3 operators for inter-operator variability, 1 operator that analyzed the datasets of nine patients three times each for intra-operator variability)	No	High
Gelaude et al. (2008)	Yes	No	No	No	High
O’Neill et al. (2012)	No	No	No	No	High
Zou et al. (2017)	No	No	No	No	High
Gangwar et al. (2018)	No	Yes	No	No	High
Väänänen et al. (2019)	Yes	Yes	No	No	Medium
Pelvis					
Zoroofi et al. (2003)	No	No	No	No	High
Anstey et al. (2011)	No	No	No	No	High
Zhou et al. (2013)	Yes	No	No	No	High
Cheng et al. (2013)	Yes	No	No	No	High
Lamecker et al. (2004)	No	No	No	No	High
Seim et al. (2008)	Yes	No	No	No	High
Kainmueller et al. (2009)	Yes	No	No	No	High
Audenaert et al. (2019)	Yes	No	No	No	High
Chu et al. (2015a)	No	No	No	No	High
Chu et al. (2015b)	No	No	No	No	High
Hanaoka et al. (2017)	No	No	No	No	High
Wang et al. (2019)	Yes	Yes	No	No	Medium
Noguchi et al. (2020)	Yes	Yes	No	No	Medium
González Sánchez et al. (2020)	No	No	No	No	High
Hiasa et al. (2020)	No	No	No	No	High
Jeuthe et al. (2021)	Yes	Yes	No	No	Medium
Liu et al. (2021)	Yes	Yes	No	No	Medium
Xu et al. (2022)	No	No	No	No	High
Wu et al. (2022)	Yes	No	No	No	High
Zhai et al. (2023)	Yes	Yes	No	No	Medium
Guo et al. (2018)	No	No	No	No	High

## 4 Discussion

The aim of the present work was to systematically review the literature on clinical CT image segmentation methods for the bones in the human hip to establish the current level of evidence on accuracy, robustness, reproducibility and repeatability, to support the use of these methods for quantifying image-based bone biomarkers in large clinical cohorts. We found that studies that reported the accuracy of segmentation of the femur and the pelvis used different metrics, making inter-study comparison challenging. However, even though there may exist a need to standardize reporting of accuracy across studies, the accuracy of automatic segmentation methods in terms of predicting ground truth manual segmentation appears to be as high, if not higher than has been achieved with semi-automated methods. This is a positive finding as automation is essential for the translation of any segmentation method to clinical practice. With respect to robustness of the automated or semi-automated segmentation methods that we reviewed, we found reporting to be sparse, with only one paper on automatic segmentation reporting robustness as a percentage of successfully completed segmentations but not reporting robustness across different scanners. Finally, we found reproducibility and repeatability generally not to be reported in the segmentation studies and only a few studies reported reproducibility in the form of inter-operator differences. Here, interestingly, but perhaps not surprisingly, one of the few studies that reported inter-operator differences found it to be larger for manual segmentation than for a semi-automatic graph cut segmentation protocol (Pauchard et al., 2016). This points to the weakness of using manual segmentation from a single rater as the ground truth when validating segmentation methods.

We believe that the present study clearly shows that further work is necessary to investigate which is the best automatic segmentation method to integrate into a clinical workflow for estimating CT-based biomarkers for femur fracture risk assessment, not only in terms of accuracy, robustness, reproducibility, and repeatability, but also in terms of computational cost. However, in the authors’ opinion, artificial intelligence-based approaches, such as deep-learning-based or machine learning-based methods, may represent the most promising methods for developing fully automated workflow for deriving CT-based biomarkers. The advantages of such methods are related to decreasing human interaction and reducing the computational cost with respect to other non-machine learning based automated methods, which are two essential requirements for clinical application of segmentation methods. This is supported by the fact that recently, AI-based methods for segmentation have been implemented into commercial software platforms (e.g. Mimics, Simpleware). However, to the authors’ knowledge, there is no work that has have evaluated the accuracy, robustness, reproducibility, and repeatability of these methods in the femur and the pelvis.

It is important to highlight that there are some limitations associated with this review. First, not all studies reported the CT imaging specifications and parameters, which might influence the segmentation results. Second, not all studies used the same metrics to assess the segmentation accuracy and they were evaluated on datasets of very different sizes and demographics, which makes inter-study comparison challenging. As such, in future studies, the use of standardized metrics, and perhaps, publicly available test datasets, would ease the comparison across methods. Moreover, not all studies reported computation time and the memory requirements for the segmentation process. This is crucial information for understanding the cost associated with acquiring the segmentations and the ease of incorporating them into clinical practice. Finally, it is important to highlight that most of the studies included in this review were limited to methods applied to non-pathological femur and/or pelvis. However, approaches successfully applied to other skeletal sites and not yet applied to femur/pelvis can be found in the literature. Some examples based on convolutional neural networks and successfully applied to vertebra are reported in (Lessmann et al., 2019; Sekuboyina et al., 2021). Due to the emergence of several artificial intelligence-based approaches applied to specific skeletal districts and also across multiple regions of interests (Mazurowski et al., 2023) the interesting point will be to evaluate if such methods are sufficiently accurate for the creation of FE models for any bone in the body.

## 5 Recommendations

Bearing in mind the need in the field of image-based bone biomarker research and clinical practice to have access to accurate, robust, reproducible and repeatable automatic methods to segment bones, we propose that future work puts emphasis on the acquisition of high-quality datasets, preferably published in an open-access repository, for advancing the development of a standardized segmentation protocol. Human donor specimens with soft tissue attached could be scanned to replicate the beam hardening effect of soft tissue in clinical scans. Thereafter, the same specimens could be scanned in high accuracy optical scanners after removal of soft tissues and the resulting hard tissue surfaces used as ground truth. This option could be useful to establish the accuracy of the segmentation methods. However, the scans should preferably be taken soon after death to maintain a similar texture and noise level as an in-vivo scan. To assess the robustness and reproducibility of segmentation methods, repeated in-vivo scans on human subjects on multiple scanners could be acquired. Moreover, to investigate repeatability, the data acquisition could include sessions of rescanning with repositioning. In the case of using in-vivo CT scans, the ground truth could be obtained by using the STAPLE method (Warfield et al., 2004), which generates ground truth data by combining (“stapling”) multiple segmentations from different expert raters, to address the inter-rater variability in manual segmentation that is used to derive the gold standard.

To correct for partial volume effect (PVE) that represents an important issue when CT images at clinical resolution are used to segment the femur, leading to errors in surface reconstruction, we suggest performing a comparison across imaging modalities of different resolutions such as micro-CT, HR-pQCT and CT with the ultimate aim to develop segmentation methods that are able to directly correct for PVE. To validate the segmentation methods, we propose the use of the HD metric for quantifying accuracy, as the ultimate goal of any segmentation method is to accurately capture the surface of the organ under investigation. Moreover, we suggest reporting HD metric in different regions i.e., femoral neck, head, and shaft. The femoral neck and head represent critical regions to be segmented due to their thin cortical thickness and thus a lower accuracy in such regions would be more critical than in the shaft, especially in view of using the segmentations in FE analysis. To quantify robustness, we propose the use of percentage of segmentations that achieved HDs that are smaller than the in-plane resolution of the image data. To evaluate the reproducibility and repeatability, CV_RMSE_ of HD may be quantified to investigate the consistency of the results using the same CT dataset, the inter- and intra-operator differences, and the effect of a re-scanning taken several days apart.

## 6 Conclusions

In conclusion, we found that automatic segmentation tools developed to date have produced at least as accurate outcomes as methods that require manual intervention. The development of automatic segmentation tools has thus matured far enough to suggest their use for quantifying image-based bone biomarkers in large clinical cohorts, as these methods can be operator independent and process images at low cost. However, only few studies systematically investigated the robustness of automatic segmentation methods, and limited data is available on their reproducibility and repeatability. These aspects require a more in-depth investigation in future studies. To this end, the development of open-access CT data and standardized metrics for quantifying accuracy, robustness, reproducibility, and repeatability in future works is recommended. Access to the CT data could be provided under the condition that methods developed or validated on the data be released as open source.

## Data Availability

The original contributions presented in the study are included in the article/Supplementary Material, further inquiries can be directed to the corresponding author.

## Appendix A

In the following, a brief description of the main segmentation methods adopted for femur/pelvis has been reported.

a) *Threshold-based method*. In this approach, the pixels are binarized depending on their intensity value. The segmentation is achieved by grouping all pixels with intensities greater than the threshold into one class and all other pixels into another class.
b) *Statistical shape model-based method*. This method aims to use prior knowledge about the shape to segment images and describes the anatomical variation observed in medical images. To create a statistical shape model, a training dataset that corresponds to a database of ground truth segmentations is used to compute the mean shape and extract the variation. The mean shape that represents the knowledge about the general shape is computed as the shape to which all other shapes in the training dataset have minimal distance to. The principal modes of variation, that corresponds to the knowledge about how much the shape differs between subjects, is extracted using principal component analysis by computing the eigenvectors of the covariance matrix. The eigenvectors corresponding to the eigenvalues of the covariance matrix are the directions of variation present in the data.
c) *Atlas-based method*. This method is based on the atlas that is assumed as a reference image and in which the region of interest has been accurately segmented, generating the binary mask. To segment a new image, the atlas is first registered to the new image, and then the binary mask is deformed from the atlas onto the new image to segment it.
d) *Graph-cut method*. This method represents an energy-based method in which an image is converted to a graph and the image segmentation problem is transformed into a cost function minimization problem. A graph of an image consists of a set of vertices and edges that connect them. Each image voxel corresponds to a vertex in the graph. Besides these vertices, there are two additional vertices, called terminal vertices that are used to represent the object and the background. Two kinds of edges can be identified: n-edges that connect two neighboring voxels, and t-edges that link each voxel to the terminal vertices. All edges in the graph are assigned some cost. The cost of n-edges corresponds to a penalty for discontinuity between the voxels, whereas the cost of a t-edge corresponds to a penalty for assigning the corresponding label to the voxel. An s-t cut is defined as a subset of edges such that the terminal vertices become completely separated. The idea of the method is to compute the best cut that would furnish the optimal segmentation. Thus, to find the optimal segmentation the cut that has the minimum cost among all cuts must be found.
e) *Deep-learning method*. This method uses deep neural networks to automatically perform the segmentation. An essential phase of such a method is the training of the networks. The most common approach in medical image segmentation is to use ground truth data to train the network to perform the desired segmentation task. For the training, it is important to use datasets that contain a variety of data that can be found in clinical practice.

## Glossary

### Anatomical abbreviations

All: union of all quadrants
FH: femoral head
FN: femoral neck
IA: inferior-anterior quadrant
IT: intertrochanter
SA: supero-anterior quadrant
SP: superior-posterior quadrant
TH: total hip
TR: trochanter

### Densitometric abbreviations

aBMD: areal bone mineral density
BMD: bone mineral density
ECTD: average density in the trabecular compartment close to the cortex
vBMD: volumetric bone mineral density
vBMD-C: cortical volumetric BMD
vBMD-I: integral volumetric BMD
vBMD-T: trabecular volumetric BMD

### Measurements and indices abbreviations

AD: average symmetric surface distance
ADE: average distance error
**AD**_**RMS**_: average symmetric roots mean square surface distance
ASD: average surface distance
BMI: body mass index
BR: buckling ratio
CM: cortical mass per unit cortical surface area
CortShaftThick: cortical thickness of shaft
CortNeckThick: cortical thickness of neck
CortArea: cortical area
CSA: cross-sectional area
CSMI: cross-sectional moment of inertia
CTh: cortical thickness
CV: coefficient of variation
%CV: percent cortical volume
**CV**_**RMS**_: root mean square coefficient variation
**d**_**cort**_: vertebral cortical thickness
**d**_**mean**_: mean distance
**d**_**r**_: area deviation
**d**_**RMS**_: root mean square distance
DS: surface distance
DSC: Dice similarity coefficient
FN Delta: the displacement between the geometric center of the mineral mass projection profile and its centre of mass
FN Sigma: standard deviation of the mineral mass projection profile
FNG: false negatives
FO: false overlap
FP: false positives
HD: Hausdorff distance
HR: Hazard Ratio
JAC: Jaccard similarity index
LTI: local thinning index
MAE: mean absolute error
MD: maximum distance
MDE: maximal distance error
ME: mean error
M-HD: modified Hausdorff distance
MO: mean overlap
NBSI: FN bending/torsional strength index
NCSI: FN compressive strength index
TCSI: trochanteric compressive strength index
NSA: neck-shaft angle
OR: odds ratio
**R** ^**2**^: coefficient of determination
RE: relative error
RMSD: root-mean-squared distance error
RMSE: root mean square error
RMS-SYM: root mean-square average symmetric distance
**r**_**tot**_: radii of the vertebral body
**r**_**trab**_: radii of the vertebral trabecular compartment
SD: standard deviation
**SD**_**RMS**_: root-mean-square standard deviation
**SSD**_**gAvg**_: symmetric surface distance global average
**SSD**_**Max**_: symmetric surface distance maximal surface distance
**SSD**_**SAvg**_ **Avg**: symmetric surface distance average of slice averages
SYM: average symmetric distance
THRCTM: ratio between cortical and trabecular mass of total hip
TN: true negatives
TP: true positives
V: volume
VD: volume difference
**VOE**_**g**_: volumetric overlap global error
**VOE**_**S**_ **Avg**: average slice error
**VOE**_**S**_ **max**: slice maximum error
**VOE**_**S**_ **min**: slice minimum error
X±SD: absolute difference mean and standard deviation

### Other abbreviations

ARF0: predicted absolute risk of current fracture (from multi-scale modelling)
CBM: cortical bone mapping
CM: cortical bone mass
DCD: directional cut discrepancy
EFFECT: European Femur Fracture Study
F: female
FL: failure load
FRAX: fracture risk assessment tool
HC: Harrells C
L1, L2 and L3: vertebral levels
M: male
MrOS: Osteoporotic Fractures in Men Study
N: number
NR: not reported
PA: pixel accuracy
PC1: principal component analysis to develop a global FE-derived risk index based on the FE parameters which were mutually correlated
SL: values were computed as means over the corresponding clusters of selected slices
SSDM: statistical shape and density modeling
Zmin: section modulus along the weakest axis
