# Supplementary material for: Segmentation methods for quantifying X-ray Computed Tomography based biomarkers to assess hip fracture risk: a systematic literature review
Source: Front Bioeng Biotechnol. 2024 Oct 23;12:1446829. doi: 10.3389/fbioe.2024.1446829 (PMC11537876; doi:10.3389/fbioe.2024.1446829)
Supplement: Supplementary file 1 [file Table1.docx]

**Table 3**: Segmentation methods developed for femur from the studies included in the review. The table shows the following information: reference of study; number of datasets N and type of material segmented; type of CT scanner, scanning parameters, and image resolution; segmentation method; metrics used to evaluate accuracy, robustness, reproducibility and repeatability; and remarks. NR: not reported.

| **Study** | **N datasets segmented, type of material** | **CT-scanner, scanning parameters and resolution** | **Metrics used for accuracy, robustness, reproducibility and repeatability** | **Remarks** |
| --- | --- | --- | --- | --- |
| **Threshold-based** | | | | |
| [Kim et al., 2018](https://www.zotero.org/google-docs/?n44en5) | 8 in-vivo CT datasets (women with no metabolic bone disorders) | CT scanner: Lightspeed VCT, GE Healthcare, Waukesha, WI, USA;  CT scanning parameters: 120 kVp, 150 effective mAs, beam collimation=20 mm, rotation speed=0.6 s, pitch=0.969:1  The calibration phantom (Mindways, Inc., Austin, TX, USA) used to convert HUs to an equivalent BMD based on the known density of K_2_ HPO_4_ in the calibration phantom  In-plane pixel size=0.6 to 0.7 mm and slice thickness=0.625 mm | Accuracy: global DSC (%), ASD (mm) and average relative error (%) of the risk factor compared with manually segmented FE models  Robustness: NR  Reproducibility: NR  Repeatability: NR | Automatic method  The method uses the complementary characteristics between the thresholding technique and the watershed algorithm  A comparison between the proposed method, snake-based semi-automated segmentation, and Simplified Kang-based automated segmentation (SK-based method) has been performed  The Authors also performed FE analyses to evaluate the effect of segmentation accuracy on the structural behavior  Manual segmentation as the ground truth  The developed method is not publicly available  Computation time: 231.6 s proposed method, 1993.9 s snake-based method, 148.1 s SK-based method, 8617.5 s manual segmentation  Computation time per slice: 1.25 ± 0.27 s proposed method, 10.75 ± 2.67 s snake-based method, 0.79 ± 0.04 s SK-based method, 45.93 ± 9.59 s manual segmentation |
| **SSM-based** | | | | |
| [Fritscher et al., 2007](https://www.zotero.org/google-docs/?aD3fvO) | 22 in-vivo CT datasets | 12 CT datasets: device and scanning parameters NR, 1.4 x1.4x1.4 mm^3^  10 CT datasets: device and scanning parameters NR, 1 dataset 120 kV, 1.4x1.4x1.25 mm^3^; 2 datasets 120 kV, 1.4x1.4x2.5 mm^3^; 1 dataset 120 kV, 1.4x1.4 x 3.75 mm^3^; 3 datasets 120 kV, 1.4x1.4x5 mm^3^; 1 dataset 140 kV, 1.4x1.4x0.6 mm^3^; 1 dataset 140 kV, 1.4x1.4x1.25 mm^3^; 1 dataset 140 kV, 1.4x1.4x2.5 mm^3^ | Accuracy: DSC, and mean Euclidian distance  Robustness: adding image artifacts which are imitating screws inside the femur and a black blob  Reproducibility: NR  Repeatability: NR | Automatic method  12 CT scans are used to build the shape-intensity model  Manual segmentation as the ground truth  The developed method is not publicly available  Computation time ∼8min |
| [Zhang et al., 2014](https://www.zotero.org/google-docs/?qtlPN0) | 41 in-vivo CT datasets, in-vivo | Device and scanning parameters NR  1.0x1.0x1.6 mm^3^ | Accuracy: RMSE of mesh fitting during the training phase (mm)  Robustness: NR  Reproducibility: NR  Repeatability: NR | Semi-automatic method  The fitting accuracy was investigated comparing the region-based SSM (rSSM) method and non-regional SSM (nrSSM) approach  The developed method is not publicly available |
| [Almeida et al., 2016](https://www.zotero.org/google-docs/?FVuGQf) | 148 femurs from in-vivo CT datasets  5 in-vivo low-resolution CT datasets (10 femurs, all electable for segmentation). These datasets are examples of the standard resolution for CT images on the lower limb for diagnosis purposes | Device and scanning parameters NR  High resolution CT datasets: - 0.666±0.069x 0.666±0.069x 0.625 mm^3^  Low resolution CT datasets: 0.781x0.781x5 mm^3^, 0.678x0.678x7 mm^3^, 0.775x0.775x5 mm^3^, 0.742x0.742x5 mm^3^, 0.742x0.742x5 mm^3^ | Accuracy: global DSC (%), ME (mm), average HD (mm)  Robustness: reported in terms of percentage of successfully completed segmentations  Reproducibility: NR  Repeatability: NR | Automatic method  Results obtained with the proposed method were compared with manual segmentation (ground truth). However, as manual segmentation of high-resolution images is very time consuming, only 10 femurs were considered for the comparative analysis.  The developed method is not publicly available  Computation time:  - for high resolution images is about 1 min  - for low resolution images the algorithm converged in 25.27 s |
| **Atlas-based** | | | | |
| [Whitmarsh et al., 2014](https://www.zotero.org/google-docs/?nSW8Nq) | 30 in-vivo CT scans of pelvic area | Siemens Sensation 64 Slice CT system (Siemens Healthcare, Erlangen, Germany)  0.7x0.7x0.5 mm^3^ | Accuracy: DSC, HD (mm), FO (mm^3^), MASD (mm)  Robustness: NR  Reproducibility: NR  Repeatability: NR | Automatic method  The atlas is constructed by manual delineations of the bones  Several common atlas combination strategies are evaluated to find one best suited for bone structures: a simple majority voting scheme, a global-weighted combination strategy, the statistical fusion method STAPLE and generalized local-weighted voting  Manual segmentation as the ground truth  The developed method is not publicly available |
| [Carballido-Gamio et al., 2015](https://www.zotero.org/google-docs/?jQ0gma) | 131 in-vivo CT scans | Three clinical facilities: 1) site A GE Light Speed QX-I, 0.742×0.742×2.5 mm^3^; 2) site B GE Light Speed 16, 0.938×0.938×2.5 mm^3^; 3) site C GE Light Speed VCT, and SIEMENS Biograph, 0.977×0.977×1 mm^3^  Scans from site A were acquired with a CT calibration phantom (Mindways Inc., Austin, TX, USA) for individual conversion of HU to equivalent reference concentrations of aqueous K_2_HPO_4_.  Scans from sites B and C were acquired with a solid CT calibration phantom (Image Analysis, Inc., Columbia, KY, USA) containing cells of 0, 75, and 150 mg/cm^3^ equivalent concentration of calcium hydroxyapatite. | Accuracy: DSC, FNG, JAC, SYM (mm), RMS-SYM (mm), M-HD (mm)  Robustness: evaluated by using 80 scans of older women from two different clinical sites and two highly anisotropic spatial resolutions (mean DSC, mean SYM (mm), mean M-HD (mm), HD (mm))  Reproducibility: NR  Repeatability: evaluated using repeated scans after repositioning from 22 subjects obtained on CT imaging systems from two manufacturers. | Automatic method  The accuracy was evaluated against manual segmentations (ground truth) of 80 scans, 50 scans from site A and 30 from site B. Manual segmentations of the other 51 scans were not available  The developed method is not publicly available |
| [Besler et al., 2018](https://www.zotero.org/google-docs/?blFWep) | 3 cadaveric CT datasets (6 femurs). All specimens displayed degraded joints due to age | GE Revolution CT Scanner (GE Healthcare)  0.684x0.684x0.625 mm^3^ | Accuracy: DSC, HD (mm)  Robustness: NR  Reproducibility: NR  Repeatability: NR | Automatic method  Developing of an automated method of segmenting femurs in clinical CT dataset with a special focus on degraded joints.  Two existing segmentation techniques using the graph-cut framework are explored for full femur segmentation (Pauchard et al. method [(Pauchard et al., 2016)](https://www.zotero.org/google-docs/?AnITee) and Krcah et al. method [(Krcah et al., 2011)](https://www.zotero.org/google-docs/?P9LHYB)). An atlas-based segmentation method using a selection criterion is proposed.  Atlas-based segmentation was conducted with and without the contralateral femur in the atlas. Thus, two atlas-based segmentations were generated for each case, one with the contralateral femur in the atlas (con.) and one without the contralateral femur in the atlas (alt.).  Manual segmentation as the ground truth  The developed method is not publicly available |
| **Graph-cut based** | | | | |
| [Krcah et al., 2011](https://www.zotero.org/google-docs/?BASpP4) | 197 CT datasets, type of material NR | Device and scanning parameters NR  Volume spacing ranged from 0.6 to 1.17 mm in-plane and from 0.8 to 1.25 mm inter-slice | Accuracy: HD (mm), TPR, FPR  Robustness: NR  Reproducibility: NR  Repeatability: NR | Automatic method  Comparison between the graph-cut method developed by the Authors and three existing fully automatic bone segmentation schemes: gradient-based geometric active contour (GeomAC), Zhang iterative adaptive thresholding (ZIAT) and intensity-based graph-cut (IBGC) methods.  To assess the accuracy, manual segmentations were used as ground truth  The developed method is publicly available.  Computation time (min): 27 for GeomAC, 20 for Ziat, 2 for IBGC, 3 for the proposed method |
| [Huang et al., 2015](https://www.zotero.org/google-docs/?sEZSLK) | 84 in-vivo CT datasets: 40 samples were selected randomly as candidates for model training, the remaining 44 femurs and 16 randomly chosen from the training dataset (for a total of 60) were used as the testing subset. The subjects included were asymptotic of any femoral diseases and the appearance of their femur was normal | 64-slice multi-detector CT scanner LightSpeed Ultra (GE Healthcare, Milwaukee, WI, USA); voltage 120 kVp, X-ray tube current of 300 mA, 0.6-s scan time, 50-cm scan field of view 0.625 mm thick slice | Accuracy: DSC, ASD (mm)  Robustness: NR  Reproducibility: NR  Repeatability: NR | Semi-automatic method  The Authors proposed the graph-cut-based segmentation with shape prior (SP-GC).  The results were compared to the active shape model (ASM) method and traditional graph cut (GC) approach, with results from manual delineation used as the ground truth.  The developed method is not publicly available. |
| [Pauchard et al., 2016](https://www.zotero.org/google-docs/?vbCyXw) | 24 in-vivo CT scans (48 femurs)  The CT datasets have been selected from normal, osteopenic and osteoporotic subjects | 4-detector CT system (Sensation, Siemens Medical Systems, Erlangen, Germany) with a bone mineral reference standard (3-sample calibration phantom, Image Analysis, Columbia, KY USA) containing calibration cells of 0, 75 and 150 mg/cm^3^  Scan parameters: 120 kVp, 140 mAs, 1-mm slice thickness, pitch = 1, 1×1×1 mm^3^ | Accuracy: HD (mm), DSC, FE results  Robustness: NR  Reproducibility: evaluation of inter-operator variability using HD (mm), maximum value of mean surface-to-surface distance, maximum value of DSC  Repeatability: NR | Semi-automatic method  The principal outcome measures for the comparison of the FE results between the two segmentation methods were whole bone stiffness (K), calculated as the slope of the greater trochanter force-displacement response up to a displacement of 0.4 mm, and peak force (F) taken as the reaction force at the greater trochanter support nodes at 4% nominal strain.  Manual segmentation as the ground truth  In the inter-operator reproducibility study, 12 left femurs have been segmented with graph cut-based method and manual segmentation by three operators ⇒ average mean surface-to-surface distance, DSC and HD were calculated between graph cut segmentations and manual segmentations from the three operators in pair-wise manner  The developed method is publicly available  Computation time:  manual segmentation 20–35 min per bone vs 2–5 minutes per bone for graph cut segmentation |
| [Besler et al., 2021](https://www.zotero.org/google-docs/?uKFJal) | 10 cadaveric CT datasets  The cadaveric dataset included advanced skeletal pathologies  10 in-vivo CT datasets  The in-vivo datasets were close in age and scan protocol parameters as required for opportunistic screening of osteoporosis | Revolution GSI, GE Health-care, Waukesha, WI, USA CT scanner  For cadaveric CT datasets: 0.691-0.792 mm (slice thickness 0.625 mm)  For in-vivo CT datasets: 0.708-0.765 mm (slice thickness 0.8 mm) | Accuracy: AD (mm), DSC, HD (mm)  Robustness: NR  Reproducibility: SD_RMS_ (CV_RMS_) for volume, density, and failure load  Repeatability: NR | Semi-automatic method  The Authors proposed an enhancing filter integrated with a graph cut segmentation technique. Multiple enhancing filters are tested in the graph cut framework. The enhancing filters used are: 1) no enhancing at all (denoted HU for raw Hounsfield units), 2) Krcah [(Krcah et al., 2011)](https://www.zotero.org/google-docs/?YqAFiX), and 3) the proposed bone and joint filter (denoted Calgary).  Inter-operator reproducibility (three operators) evaluated by measuring the SD_RMS_ (absolute units) and CV_RMS_ (%) for volume, integral density, and failure load in both cadaveric and in-vivo CT images  The developed method is not publicly available |
| Aldieri et al., 2024 | 10 patients selected from the HipOp registry in Rizzoli Orthopaedic Institute (10 femurs, in-vivo CT)  The subjects involved were all post-menopausal women  Two of them did experience a hip fracture after the CT scan | Device NR  Tube voltage at 120 kVp, tube current at 150–200 mA and a focal spot of 0.7 mm. The slice thickness of the acquired images ranged from 2.5 to 3.0 mm and the voxel spacing (isotropic in the other two directions) was between 0.66 and 0.78 mm. | Accuracy: Difference union ratio  DUR, HD (mm), average Housdorff distance AHD (mm), blind visual comparison performed by 4 experts, FE-based fracture risk assessment (ARF0)  Robustness: NR  Reproducibility: NR  Repeatability: NR | Semi-automatic method  Each femur had been segmented four different times with both the manual and semi- automated method  Distance metrics (DUR, HD, AHD) were computed within the repeated manual and semi-automatic segmentations (intra-segmentation) as a variability measure of and across the semi-automated and manual segmentations (inter-segmentation), considering all possible pairings. The Mann–Whitney U-test was conducted to identify significant differences between the intra-segmentation and inter-segmentation metrics. The same test was also employed to compare the ARF0 values obtained starting from the segmentations output by the two methodologies  The blind visual comparison has been performed by 4 operators who compared the segmentations superimposed to the corresponding CT image slice by slice and established, if possible, the best segmentation based on its fidelity to the CT bone contour. This visual comparison was carried out blindly, aiming to avoid biases, and was conducted using a custom-made software. Since each femur had been segmented 4 different times with both the manual and semiautomated method, a representative segmentation was obtained from the four available for each methodology using the STAPLE algorithm. Besides, the blind visual comparison was limited to the proximal region of femur.  The blind evaluation analysis consisted in labelling the CT slices as Manual, Semi-automated or None, according to that which segmentation was judged the best by the operator, for all the operators involved in the analysis. The assessments were merged using a majority voting procedure, i.e., considering the frequency of each label for each patient. The frequency distributions of the labels were obtained for each patient and compared using the one-sided Wilcoxon test.  Manual segmentation as the ground truth  The developed method is publicly available  Computation time: 10-20 min |
| **Convolutional neural network** | | | | |
| [Chen et al., 2019](https://www.zotero.org/google-docs/?NUoKnj) | 150 in-vivo CT datasets (300 femurs) | CT scanner (Siemens Sensation Open 128-slice CT scanner; Siemens, Erlangen, Germany) in PLA General Hospital, China  Intra-slice resolutions ranged from 1.32 to 1.85 mm and the inter-slice resolutions had a constant value of 3.00 mm | Accuracy: DSC (%), DS (mm)  Robustness: NR  Reproducibility: NR  Repeatability: NR | Automatic method  The novelty of approach lies in two feature enhancement modules, including the edge detection task and the multiscale feature fusion  The set of 150 patients were randomly divided into two sets with 120 patients as training data and the other 30 patients as testing data. During the training, 90% data (108 patients) was used to train the model parameters, and 10% data (12 patients) was used as held-out validation data  Manual segmentation as the ground truth  The developed method is not publicly available  Computation time: 0.93 s per CT volume |
| [Yosibash et al., 2020](https://www.zotero.org/google-docs/?ODLuKZ) | 39 in-vivo CT datasets (70 femurs). The CT acquisitions were performed at different hospitals | Philips scanners (model names: Brilliance 64, Ingenuity Core 128, iCT 256, Mx8000 IDT 16)  Resolution of 512×512×320 voxels | Accuracy: DSC, ASD (mm)  Robustness: NR  Reproducibility: NR  Repeatability: NR | Automatic method  The accuracy of the automatic segmentation was checked on 41 bones (which did not participate in the training process), of male and female, young and old patients, by comparison to manual segmentation (ground truth).  The developed method is not publicly available  The average segmentation time was 12 minutes: 2.5 min to load files, cut them, align them and find FSCV, 2.5 min for running Net25, 5 min for running Net51, 2 min for postprocessing. |
| [Hiasa et al., 2020](https://www.zotero.org/google-docs/?4XXEsH) | 20 in-vivo CT volumes scanned (Osaka University Hospital THA dataset) | Device and scanning parameters NR  Field of view 360 × 360 mm^2^, matrix size 512 × 512  Slice intervals: 2.0 mm for the region including the pelvis and proximal femur, 6.0 mm for the femoral shaft region, and 1.0 mm for the distal femur region | Accuracy: DSC (%), AD (mm)  Robustness: NR  Reproducibility: NR  Repeatability: NR | Automatic method  The Osaka University Hospital THA dataset was used for training and cross-validation for the accuracy evaluation and prediction of the DSC coefficient  Manual segmentation as the ground truth  The developed method is not publicly available  Average training time: 11 hours  Average computation time for the inference on one CT volume with about 500 2D slices was approximately 2 minutes excluding file loading, and the post-processing took about 3 minutes |
| [Zhao et al., 2021](https://www.zotero.org/google-docs/?xx5Wix) | 216 W, 181 M (total=397 CT datasets, 10% for external testing), in-vivo | QCT Siemens Sensation 64, 120kVp, convolution kernel B30s, 512x512 matrix  Pixel size 0.742-0.977 mm, 2 mm slice thickness (converted to 3 mm thickness for comparison with previous FE studies - Fourier interpolation & decimation: 3x2 mm ⇒ 6x1 mm ⇒ 2x3 mm) | Accuracy: DSC, Sensitivity, Specificity, HD (mm), ASD (mm)  Robustness: evaluated performing the data augmentation since the sample size was insufficient to train a precise 3D segmentation model  Reproducibility: NR  Repeatability: NR | Automatic method  Trained male and female cohort separately, then together. V-Net for segmentation and spatial transformation (ST) for refinement - to improve results at neck region due to 3 mm slice thickness. V-Net alone results also reported.  Manual segmentation as the ground truth  The developed method is not publicly available |
| [Patton et al., 2021](https://www.zotero.org/google-docs/?DUy7Rq) | 28 cadaveric right proximal femurs from adults with no observable or known musculoskeletal trauma or pathologies | Nano-CT (proximal femurs): phoenix nanotom-s, GE Measurement & Control 110 kV, 200 μA, 0.07 mm thick brass filter, 1 skips, 3 averages, 1000 images/360 degrees.  Isotropic voxel: 27x27x27 μm^3^ | Accuracy: DSC  Robustness: NR  Reproducibility: NR  Repeatability: NR | Automatic method  The masks resulting from the method proposed by the Authors were compared to those segmented using global or local thresholding methods. Two global threshold methods: Otsu minimum two-class variance and Yen’s threshold method. One local threshold method: local Otsu method  Fully-connected CNNs were trained separately for the femoral neck and the vertebral body, and also combined.  FN U-net ⇒ U-net using only femoral neck images  VB U-net ⇒ U-net using only vertebral bodies images  FN+VB U-Net ⇒ U-net using both type of images  Manual segmentation as the ground truth  The developed method is not publicly available |
| [Deng et al., 2022](https://www.zotero.org/google-docs/?ivzjG0) | 100 in-vivo CT datasets | CT scanner manufacturer NR  CT scanning parameters were 120 kV, 250 mAs, a 50 cm field of view, standard reconstruction kernel with filtered back-projection 512×512 matrix, 1 mm reconstructed slice thickness | Accuracy: DSC, ASD (mm), TNR, TPR, MAE (cm^3^), RMSE (cm^3^), RE (%) (min, mean, max)  Robustness: data augmentation  Reproducibility: NR  Repeatability: NR | Automatic method  85 subjects were used in tenfold cross-validation for training and internal validation and to select the optimal parameters of the proposed models. The rest of the subjects (15) were used to evaluate the performance of models.  Manual segmentation as the ground truth  The developed method is not publicly available  Computation time: 0.31 s was required to segment a CT volume |
| [Zhang et al., 2022](https://www.zotero.org/google-docs/?LPAe9T) | 30 in-vivo X-ray images of femurs (20 for training sets, 5 for validation, and 5 for testing) | Device and scanning parameters NR  The slice layer thickness of the training set is between 0.4 and 0.9 mm, and the difference is relatively small. The size of each layer of data is 512×512. | Accuracy: DSC, PA, TPR, TNR  Robustness: NR  Reproducibility: NR  Repeatability: NR | Automatic method  The number of slices varies from around one hundred to around seven hundred  Manual segmentation as the ground truth  The developed method is not publicly available |
| [Kuiper et al., 2022](https://www.zotero.org/google-docs/?750Y2P) | 50 in-vivo CT scans of the lower extremity for the training and initial evaluation of the networks  10 additional in-vivo CT scans to evaluate the robustness of the final deep learning segmentation network | The 50 CT scans were acquired with either the Philips iCT scanner or  Philips Brilliance 64 (Philips Medical Systems, Best, The Netherlands; tube voltage=120 kVp, tube current=31–347 mA, effective dose=35–150 mAs, slice thickness=1 mm, slice increment=0.7 mm, pixel spacing=0.63–0.98 mm, matrix size=512×512 pixels.  The 10 CT datasets Philips Brilliance Big Bore scanner (Philips Medical Systems). tube voltage=120 kVp, tube current=82–245 mA, effective dose=100–301 mAs, slice thickness=1 mm, slice increment=0.5 mm, pixel spacing=0.63–1.17 mm, matrix size=512 × 512 pixels | Accuracy: DSC, mean HD (mm), mean MASD (mm), mean HD95 (mm), HD (mm), MASD (mm), HD95 (mm)  Robustness: evaluated using CT datasets different from the CT datasets used for training and evaluation. These CT datasets were characterized by different subject demographics and acquisition parameters (mean MASD (mm), HD (mm))  Reproducibility: NR  Repeatability: NR | Automatic method  The proposed method led to a significant improvement over the results of the state‐of‐the‐art nnU‐net, with only approximately 1/12th of training time, 1/3th of inference time and 1/4th of GPU memory required  Manual segmentation as the ground truth  The developed method is not publicly available |
| [Bjornsson et al., 2023](https://www.zotero.org/google-docs/?WyeCrq) | Sample I: 24 in-vivo CT datasets (48 femurs)  Sample II: 54 (training), 6 (validation), 1147 (testing), all in-vivo | Device and scanning parameters NR  0.977x0.977x1.0 mm^3^ | Accuracy: DSC, HD95 (mm), FE strength results (R^2^, RMSE (N), MAE (%), max difference (%))  Robustness: evaluated using two different samples  Reproducibility: NR  Repeatability: NR | Automatic method  Sample I: computation time 9 s vs 120-300 of Pauchard et al. method [(Pauchard et al., 2016)](https://www.zotero.org/google-docs/?AIRaEK)  Sample II: computation time 11 s  Manual segmentation as the ground truth  The developed method is not publicly available |
| [Apivanichkul et al., 2023](https://www.zotero.org/google-docs/?LMCT9M) | 120 CT scans of the lower abdomen of 120 patients with lower abdominal diseases including colorectal cancer, rectum cancer, prostate cancer, cervical cancer, rectosigmoid cancer | CT slices were acquired using SOMATOM Confidence 32-slice CT simulator (Siemens, Germany) in the HELIX operation (helical scanning mode) with 120 kV, 250 mA, and 3 mm slice thickness | Accuracy: highest DSC  Robustness: NR  Reproducibility: NR  Repeatability: NR | Automatic method  120 CT scans: 72 CT scans were used for training, 24 CT scans for validation, and 24 CT scans for testing  Manual segmentation as the ground truth  The developed method is not publicly available |
| Tan et al., 2024 | 41 in-vivo CT datasets (21 training set and 20 test set) | Device NR  Each CT volume consists of 111 ∼ 286 slices of 512 × 512 pixels, with a voxel spatial resolution of ([0.29 ∼ 0.67] × [0.29 ∼ 0.67] × [0.70 ∼ 1.25]) mm^3^ | Accuracy: DSC, HD95  Robustness: NR  Reproducibility: NR  Repeatability: NR | Automatic method  The type of ground truth is not explicitly mentioned  The developed method is not publicly available |
| Sultana et al., 2024 | In-vivo CT of 18 anonymous adults (10 male and 8 female) | SIEMENS S5VB40B CT scanner (Siemens Medical Solution, Malvern, USA) with acquisition and reconstruction parameters of 120 kVp and 244 mAs  In-plane resolution of 512 × 512 pixel array with approximately 1 × 1 × 1 mm3 of voxel size | Accuracy: Mean Intersection over Union (mean IoU), DSC, average precision, sensitivity, and specificity  Robustness: NR  Reproducibility: NR  Repeatability: NR | Automatic method  The performance of the method was evaluated with a testing dataset of 20 femurs belonging to 10 patients. Four among these 10 patients were known to the 3D U-Net segmentation model through validation process (validated dataset), and the other 6 patients were completely new, unseen to our segmentation model, and independent of training and validation process.  Manual segmentation as the ground truth  The developed method is not publicly available |
| Zhang et al., 2024 | 100 participants (50 females) | An ultra-low dose (ULD) hip CT protocol and a clinical standard protocol used to acquire images  All CT imaging performed on a Siemens SOMATOM Force (Forchheim, Germany) scanner  Clinical hip CT imaging was performed using single x-ray source spiral acquisition mode with the following parameters: 120 kV, 100 effective mAs, pitch factor: 0.8, scan length: 15 cm, collimation: 192 × 0.6 mm  ULD CT imaging was performed using single x-ray source spiral acquisition mode with tin (Sn) filtration and following parameters: Sn100 kV, 200 effective mAs, pitch factor: 1.0, scan length: 15 cm, collimation: 192 × 0.6 mm | Accuracy: DSC, vBMD stratified by participants’ BMI  Robustness: NR  Reproducibility: NR  Repeatability: DSC, vBMD stratified by participants’ BMI (concordance correlation coefficient (CCC) and root-mean- square coefficient of variation (RMSCV)) | Automatic method  ULD hip CT imaging was performed on all participants. Among these participants,12 participants (5 females) consented to an additional hip CT scan using a clinical protocol and another 5 participants (5 females) consented to a repeat ULD hip CT scan.  The data from human participants were divided into two subsets. The first subset included hip CT data from 20 participants (10 females), which also included clinical hip CT scans of 12 participants and repeat hip ULD CT scans of 5 participants. This dataset was used to evaluate different performance metrics. The second subset included data from the remaining 80 participants (40 females), which was used for deep learning training. All additional clinical CT and repeat ULD hip CT scans were performed after repositioning the participant on the scanner table immediately following the initial or baseline ULD scan.  The deep learning network was trained using ULD hip CT images from the 80 participants, which was randomly partitioned into learning (n = 60) and validation (n = 20) data sets. The deep learning network required approximately 36 h to complete the training phase.  vBMD measurement: for each clinical CT image, one hundred spherical regions-of interest (ROIs), each of diameter 11.5 mm, equivalently 23 voxels, were randomly selected inside the segmented femur region. Matching ROIs for an ULD CT image were generated after registering the ULD CT image with the corresponding clinical CT image.  Manual segmentation as the ground truth    The developed method is not publicly available |
| Saillard et al., 2024 | MEKANOS cohort: 11 in-vivo CT-scans of hips (both femurs present). A few femurs have metastatic osteolytic lesions 🡺 allowed trained models to segment metastatic bones more efficiently  As a secondary test dataset, additional femurs (n = 16, 9 patients) from four different centers were added from MEKANOS cohort a posteriori | MEKANOS cohort: constant table height, quality phantom QA Mindways, 120 kV, 270 mAs, 1 Pitch, Field of view 360 mm and 200 mm, reconstruction: standard filter B, 512 × 512 matrix, slice thickness 0.7 mm and 3 manufacturers acquisition systems (General Electric, Philips and Siemens) | Accuracy: DSC, HD (mm), FE-based failure load estimates  Robustness: NR  Reproducibility: Inter-operator variability (4 operators) on 6 ex-vivo femurs in terms of segmentations and FE-based failure load estimates  Repeatability: NR | Automatic method  Manual segmentation as the ground truth:  - MEKANOS cohort: 18 femurs manually segmented (4 femurs not available)  - secondary test dataset: 16 femurs manually segmented  Among the 18 available femurs, 12 were used for training, 4 for validation and 2 for testing  On 12 femurs the FE-based failure loads computed using automatic and manual segmentations  Data augmentation, such as random rotations, translations, shearing and scaling used on- the-fly to prevent overfitting  For femur segmentation, a fully automated segmentation method with a pre-processing pipeline has been proposed. The pre-processing pipeline consists of several steps such as data selection resampling, crop volumes, split volumes, flip left femurs, affine registration and intensity normalization  The developed method is not publicly available |
| **Other methods** | | | | |
| [Testi et al., 2001](https://www.zotero.org/google-docs/?OHvmgR) | 8 CT datasets: one composite femur, one in-vivo CT dataset and six in-vivo CT datasets for patients in need of a CMP | High Speed Advantage CT scanner (General Electric, USA)  Composite femur: 0.55x0.55 mm^2^, 1 mm slice thickness and 1 mm slice distance  In-vivo CT dataset: 0.42x0.42 mm^2^ | Accuracy: ME (mm)  Robustness: NR  Reproducibility: contour extraction reproducibility (RMSE of HD)  Repeatability: NR | Automatic method  The Authors developed the border-tracing method. They compared this method to the threshold-based and manual methods.  Accuracy: comparison among physical dimensions taken from composite femur with a caliper and the same measurements extracted by the contours traced using the three methods  The developed method is not publicly available |
| [Kang et al., 2003](https://www.zotero.org/google-docs/?86JTuX) | - ESP CT dataset  - Pelvic in-vivo CT datasets (9 patients) | - ESP: Somatom Sensation 16 CT scanner (Siemens, Erlangen, Germany), 120 kV, 330 mAs, collimation 0.75 mm, 0.2 x 0.2 x 0.5 mm^3^  - Pelvic CT: NR | Accuracy: r_tot_ (mm), r_trab_ (mm), d_cort_ (mm)  Robustness: Noise sensitivity study on the ESP CT dataset and femur CT images. Evaluation of noise on accuracy of r_tot_ (mm), r_trab_ (mm), d_cort_ (mm)  Reproducibility: Intra-observer variability (CV_RMS_ (%)) and Inter-observer variability (CV_RMS_ (%))  Repeatability: NR | Automatic method  Automatic region-growing technique augmented by manual correction  Segmentation accuracy was determined using the ESP: the accuracy for the radii of the vertebral bodies and their trabecular compartments as well as for cortical thicknesses was investigated comparing the values obtained from segmented images and the expected values.  Intra-observer variability: one operator analyzed each of the nine data sets three times. For each patient first the CV was calculated from the three measurements. Then the CV_RMS_ was computed.  Inter-observer variability: three operators analyzed each data set once, blinded to the results of the other operators  The developed method is not publicly available |
| [Gelaude et al., 2008](https://www.zotero.org/google-docs/?riMMwJ) | 9 CT datasets formalin-fixed cadavers (15 femurs segmented)  After removal of soft tissues, 15 femurs were re-scanned (dry femurs) | SOMATOM Sensation spiral CT scanner (Siemens AG, Erlangen, Germany)  1x1x1 mm^3^ | Accuracy: comparison with respect to ground truths $\underline{X}$±SD (mm)  Robustness: NR  Reproducibility: NR  Repeatability: NR | Automatic method  Self-developed contour-based algorithm  The study incorporates the effect of soft tissue presence on hard tissue segmentation and simultaneously reveals the accuracy shift introduced as a result of boiling the cadaver bones by processing extra CT scans of the dried bones.  Femurs without soft tissues were scanned with optical 3D scan and the STL were generated and used as ground truths.  The developed method is not publicly available. |
| [O’Neill et al., 2012](https://www.zotero.org/google-docs/?B2x5C5) | 6 in-vivo CT datasets from patients with femoral-acetabular impingements | GE Lightspeed Plus CT scanner  0.68 x 0.68 x 1.25 mm^3^ | Accuracy: VOE_g_ (%),VOE_S_Avg (%),VOE_S_min (%),VOE_S_max (%), SSD_gAvg_ (mm), SSD_sAvg_Avg (mm), SSD_Max_ (mm)  Robustness: NR  Reproducibility: NR  Repeatability: NR | Semi-automatic method  The accuracy of segmentation method proposed by the Authors (i.e. morphological snake method) was measured numerically in terms of the intersection of voxels covered by both models (proposed method and manual segmentation) and the distance from the bordering voxels in the ground truth model to the nearest bordering voxels in the model segmented through Auhors’ method.  Manual segmentation as the ground truth  The developed method is not publicly available  Computation time: 5–10 min |
| [Zou et al., 2017](https://www.zotero.org/google-docs/?4KuRMj) | 20 in-vivo CT datasets (40 femurs) from patients needing medical treatment such as total hip replacement. The work focused on more complication CT data with a narrow joint space | Device and scanning parameters NR  0.65x0.65x1.00 mm^3^ | Accuracy: JAC (%), DSC (%), DCD (mm)  Robustness: NR  Reproducibility: NR  Repeatability: NR | Semi-automatic method  The Authors proposed a semi-automatic segmentation framework based on harmonic fields and they compared their methods to Morphological Snakes (MS) based method, Level Set (LS) based method and Shape Prior Graph Cut (SPGC) based method  Manual segmentation as the ground truth  The developed method is not publicly available  In all groups, computation time per slice is lower for the proposed method (5 s) than the other methods. |
| [Gangwar et al., 2018](https://www.zotero.org/google-docs/?1r4dqf) | 11 in-vivo CT datasets (22 femurs) | 10 datasets were obtained with a Siemens SOMATOM Denition Edge (Germany): 120 or 140 kVp, 300 or 350 mAs, 0.703125x0.703125x2.5 mm^3^    One dataset was obtained with a clinical Siemens SOMATOM Sensation 64 CT (Germany): 140kVp, 350mAs, 0.703125x0.70312x2.5 mm^3^ | Accuracy: DSC, TPR, TNR    Robustness: NR  Reproducibility: NR  Repeatability: NR | Automatic method  Variational segmentation methodology  Approach for segmenting CT bone objects in the presence of thin cartilage regions  Manual segmentation as the ground truth  The developed method is not publicly available |
| [Väänänen et al., 2019](https://www.zotero.org/google-docs/?75CNba) | 13 in-vivo CT datasets. Five of the patients had a previous hip fracture on one side  17 ex-vivo (cadaver) CT datasets. 14 CT datasets were additionally imaged with a μCT system. None of the cadavers had any pre-existing conditions known to affect bone metabolism. | In-vivo CT datasets: clinical CT system Philips Precedence 6P, tube voltage 120 kV, tube current 100 mAs, in-plane resolution between 0.7 and 0.8 mm, slice separation 2.0 mm  Ex-vivo CT datasets: Siemens Somatom AS clinical CT-scanner, tube voltage 120 kV, tube current 210 mAs and CTDIvol ∼16. 14 CT datasets reconstructed using medium or soft kernels, 3 CT datasets reconstructed with hard kernel, pixel size of 0.4–0.5 mm, slice separation of 0.6 mm  Ex-vivo μCT datasets: μCT system Nikon XT H 225 scanner, 200 mAs, 100 kVp, isotropic voxel size of 0.052–0.060 mm | Accuracy: DSC, ASD (mm), VD (cm3), FE strains (R^2^, NRMSE (%), slope, intercept)  Robustness: proved by testing the automatic segmentation method on in-vivo and ex-vivo CT datasets which were acquired using different CT scanners and with different scanning parameters  Reproducibility: NR  Repeatability: NR | Automatic segmentation method in combination with Stradwin to segment the femoral periosteal and endocortical surfaces  The validation was performed both using in-vivo and ex-vivo dataset. For the in-vivo datasets, the automatic segmentations of the clinical CT images were compared to the segmentations of the same clinical CT images as performed via conventional semi-automatic segmentation using Mimics. The ex-vivo datasets consisted of a set of cadaver femurs imaged both with clinical CT and μCT. The μCT images were used as gold standard. For the ex-vivo dataset, the automatic segmentations of the clinical CT images were compared to the manual segmentations of the corresponding μCT-images.  For 3 femurs ex-vivo, FE analyses mimicking stance load were automatically formed from the automatically generated FE models. These three femurs were earlier tested experimentally in single-leg-stance loading until failure, with simultaneous recording of the surface deformations using the DIC technique. The predicted surface strains from the FE analyses were thus compared via robust linear regression analysis to the experimental measurements to evaluate the accuracy of the automatically generated FE models  The developed method is publicly available |
