# Supplementary material for: Segmentation methods for quantifying X-ray Computed Tomography based biomarkers to assess hip fracture risk: a systematic literature review
Source: Front Bioeng Biotechnol. 2024 Oct 23;12:1446829. doi: 10.3389/fbioe.2024.1446829 (PMC11537876; doi:10.3389/fbioe.2024.1446829)
Supplement: Supplementary file 2 [file Table2.docx]

**Table 4**: Segmentation methods developed for femur from the studies included in the review. The table shows the quantitative results obtained from each study for evaluating accuracy, robustness, reproducibility and repeatability. NR: not reported.

| **Study** | **Accuracy** | **Robustness** | **Reproducibility** | **Repeatability** |
| --- | --- | --- | --- | --- |
| **Threshold-based** | | | | |
| [Kim et al., 2018](https://www.zotero.org/google-docs/?n44en5) | Global DSC (%): 97.24±0.44 for the proposed method, 98.02±0.36 for snake-based, 96.08±1.94 for SK-based  ASD (mm): 0.36±0.07 for the proposed method, 0.26±0.06 for snake-based, 0.50±0.24 for SK-based  Average relative error (%) of the risk factor compared with manually segmented FE models: 4.99 for the proposed method, 3.51 for snake-baed method and 11.61% for SK-based method | NR | NR | NR |
| **SSM-based** | | | | |
| [Fritscher et al., 2007](https://www.zotero.org/google-docs/?aD3fvO) | DSC: 0.992±0.006  Mean Euclidian distance: 0.20 mm± 0.063 | The accuracy of the segmentation is equal to the segmentation result of the same datasets without artifacts | NR | NR |
| [Zhang et al., 2014](https://www.zotero.org/google-docs/?qtlPN0) | RMSE of mesh fitting during the training phase (mm): head 0.30±0.069, great trochanter 0.42±0.079, proximal shaft 0.42±0.062, distal shaft 0.40±0.045, lateral condyle 0.43±0.087, medial condyle 0.44±0.066, whole femur 0.52±0.091, non-regional SSM 0.62±0.21 | NR | NR | NR |
| [Almeida et al., 2016](https://www.zotero.org/google-docs/?FVuGQf) | High resolution CT datasets:  - DSC (%) 94±1.6  - ME (mm) 1.014±0.474  - Average HD (mm) 4.336±0.861  Low resolution CT datasets:  - ME (mm) 1.446±1.101  - Average HD (mm) 10.135±5.948 | Only 3 failed to converge, therefore the success rate is 98%, in spite of the significant variability of gender and age- related bone loss. | NR | NR |
| **Atlas-based** | | | | |
| [Whitmarsh et al., 2014](https://www.zotero.org/google-docs/?nSW8Nq) | DSC: - Femur: 0.970 ± 0.023, 0.984 ± 0.004, 0.985 ± 0.004, 0.982 ± 0.005, 0.988 ± 0.002, for Single Atlas, Majority voting, weighted voting, STAPLE and Local weighted voting strategies respectively  - Pelvis: 0.958±0.006, 0.977±0.004, 0.978±0.003, 0.975±0.005, 0.981±0.005 for Single Atlas, Majority voting, weighted voting, STAPLE and Local weighted voting strategies respectively  HD (mm): - Femur: 4.83±3.17, 2.99±2.07, 2.85±2.03, 2.92±2.10, 2.98±0.99 for Single Atlas, Majority voting, weighted voting, STAPLE and Local weighted voting strategies respectively  - Pelvis: 6.02±3.01, 3.59±2.33, 3.58±2.23, 4.00±2.71, 2.98±2.24 for Single Atlas, Majority voting, weighted voting, STAPLE and Local weighted voting strategies respectively  FO (mm^3^): - Femur-pelvis: 161.9±219.4, 63.3±50.1, 62.9±49.2, 68.3±55.4, 40.6±48.1 for Single Atlas, Majority voting, weighted voting, STAPLE and Local weighted voting strategies respectively  MASD (mm): - Femur: 0.49±0.33, 0.29±0.07, 0.28±0.06, 0.32±0.09, 0.23±0.04 for Single Atlas, Majority voting, weighted voting, STAPLE and Local weighted voting strategies respectively  - Pelvis: 0.46±0.06, 0.27±0.04, 0.27±0.04, 0.31±0.06, 0.24±0.06 for Single Atlas, Majority voting, weighted voting, STAPLE and Local weighted voting strategies respectively | NR | NR | NR |
| [Carballido-Gamio et al., 2015](https://www.zotero.org/google-docs/?jQ0gma) | DSC 0.976±0.006  FNG 0.030±0.010  JAC 0.953±0.011  SYM (mm) 0.203±0.057  RMS-SYM (mm) 0.521±0.135  M-HD (mm) 0.219±0.071 | Mean DSC 0.976  Mean SYM (mm) 0.203  Mena M-HD (mm) 0.253  HD (mm) 3.928 | NR | - CV_RMS_ (%): 0.89 for mean integral femur vBMD, 0.96 for Mean integral head vBMD, 1.27 for Mean integral neck vBMD, 0.96 for Mean integral trochanter vBMD, 1.34 Mean trabecular bone neck and trochanter vBMD, 1.04 for Mean cortical bone neck and trochanter vBMD, 1.66 for Mean trabecular bone neck vBMD, 1.33 for Mean cortical bone neck vBMD, 1.30 for Mean trabecular bone trochanter vBMD, 1.02 for Mean cortical bone trochanter vBMD;  - CV_RMS_ (%): 0.20 for Integral femur volume, 0.27 for Integral neck and trochanter volume, 0.98 for cortical neck and trochanter volume, 0.44 for Integral neck volume, 1.82 for Cortical neck volume, 0.26 for Integral trochanter volume, 1.05 for Cortical trochanter volume;  - CV_RMS_ (%) 3.51 for FE strength in stance and 3.59 for FE strength in fall;  - CV_RMS_ (%): 2.03 for neck and trochanter thickness, 2.69 for neck thickness, 1.89 for trochanter thickness;  CV_RMS_ (%): 1.30 for neck and trochanter mean laminar vBMD, 2.19 for neck mean laminar vBMD, 1.08 for trochanter mean laminar vBMD |
| [Besler et al., 2018](https://www.zotero.org/google-docs/?blFWep) | DSC: 0.994 for Pauchard et al. method [(Pauchard et al., 2016)](https://www.zotero.org/google-docs/?6zqsfP), 0.801 for Krach et al. method [(Krcah et al., 2011)](https://www.zotero.org/google-docs/?QPXEf6), 0.978 for the proposed method (con.), 0.969 for the proposed method (alt.)  HD (mm): 4.58 mm for Pauchard et al. method [(Pauchard et al., 2016)](https://www.zotero.org/google-docs/?Lp3pQZ), 102.40 mm. for Krcah et al. method [(Krcah et al., 2011)](https://www.zotero.org/google-docs/?gGHNxB), 5.95mm for the proposed method (con.), 8.53mm for the proposed method (alt.) | NR | NR | NR |
| **Graph-cut based** | | | | |
| [Krcah et al., 2011](https://www.zotero.org/google-docs/?BASpP4) | For the proposed method: HD<8 mm, TPR>0.85, FPR<0.001  For GeomAC, ZIAT and IBGC methods HD>5 cm | NR | NR | NR |
| [Huang et al., 2015](https://www.zotero.org/google-docs/?sEZSLK) | DSC: 0.96±0.0130 for SP-GC, 0.8769±0.0539 for ASM and 0.9358±0.0150 for GC  ASD (mm): 0.885±0.933 for SP-GC, 2.148±1.783 for ASM, 1.154±1.438 for GC | NR | NR | NR |
| [Pauchard et al., 2016](https://www.zotero.org/google-docs/?vbCyXw) | HD (mm) 3.75±1.26 with absolute minimum and maximum values of 2.18 mm and 9.0 mm, respectively.    DSC 0.973±0.005, ranging from 0.963 to 0.981.  FE results: The Bland-Altman plots did not reveal any particular trends in the difference between the two segmentation methods. Average absolute difference in stiffness was −3.4% (max difference 26.6%) and in peak force −3.4% (max difference 18.5%). | NR | Inter-operator variability ⇒ maximum value of HD 3.09 mm for manual segmentations vs. 3.49 mm for graph-cut segmentations; maximum value of mean surface-to-surface distance -0.378 mm for manual segmentations vs. 0.006 mm for graph-cut segmentations; maximum value of DSC 0.980 for manual segmentations vs. 0.995 for graph-cut segmentations | NR |
| [Besler et al., 2021](https://www.zotero.org/google-docs/?uKFJal) | Cadaveric CT datasets:  AD_HU_ (mm)=0.59 (0.24-0.73)  AD_Krcah_(mm)=0.32 (0.23-0.54)  AD_Calgary_ (mm)= 0.30 (0.22-0.45)  DSC_HU_=0.96 (0.95-0.98)  DSC_Krcah_=0.97 (0.96 -0.98)  DSC_Calgary_=0.98 (0.97 -0.98)  HD_HU_ (mm)=9.20 (2.83-15.00)  HD_Krcah_(mm)=3.16 (3.00-6.40)  HD_Calgary_ (mm)=4.73 (2.24-7.62)  In-vivo CT datasets:  AD_HU_ (mm)=0.65 (0.36-0.85)  AD_Krcah_(mm)=0.36 (0.29-0.42)  AD_Calgary_ (mm)=0.31 (0.25-0.38)  DSC_HU_=0.96 (0.94-0.97)  DSC_Krcah_= 0.97 (0.97-0.98)  DSC_Calgary_=0.97 (0.97-0.98)  HD_HU_ (mm)=12.19 (2.24-16.43)  HD_Krcah_(mm)=3.00 (2.24-5.39)  HD_Calgary_ (mm)=2.24 (2.00-3.46) | NR | SD_RMS_ (CV_RMS_) for volume, density, and failure load was 9.58 ml (5.41%), 2.02 mg/cc (0.65%), and 70.10 N (5.17%) for in-vivo CT datasets, 7.26 ml (4.10%), 1.86 mg/cc (0.92%), and 34.10 N (6.43%) for cadaveric CT datasets | NR |
| Aldieri et al., 2024 | DUR: 0.06 ± 0.02  HD (mm): around 4  Average Housdorff distance AHD (mm): 0.03 ± 0.01  Blind visual comparison: the median of the distribution corresponding to the Semi-automated label significantly higher than the other two (p-value < 0.001), with no significant difference between the Manual and the None distributions.  The ARF0 assessed in silico starting from both segmentations showed no significant difference emerged between the two segmentations (R^2^ = 0.99). | NR | NR | NR |
| **Convolutional neural network** | | | | |
| [Chen et al., 2019](https://www.zotero.org/google-docs/?NUoKnj) | Proposed method:  DSC (%): 96.88±0.95  DS (mm): 0.41±0.11  Fully convolutional network (FCN) without multi-scale feature fusion:  DSC (%): 94.13±1.76  DS (mm): 0.71±0.16  Fully convolutional network (FCN) without edge detection task:  DSC (%): 96.04±1.03  DS (mm): 0.53±0.14 | NR | NR | NR |
| [Yosibash et al., 2020](https://www.zotero.org/google-docs/?ODLuKZ) | DSC: 0.98±0.003  ASD (mm): 0.36±0.05 | NR | NR | NR |
| [Hiasa et al., 2020](https://www.zotero.org/google-docs/?4XXEsH) | DSC (%): 98.5±0.65  AD (mm): 0.175±0.084 | NR | NR | NR |
| [Zhao et al., 2021](https://www.zotero.org/google-docs/?xx5Wix) | ST-V-Net  - F+M: DSC=0.9888±0.0047  Sensitivity=0.9966±0.0013  Specificity=0.9988±0.0001  HD (mm)=5.917±1.412  ASD (mm)=0.009±0.004  - M: DSC=0.9911±0.0033  Sensitivity=0.9947±0.0016  Specificity= 0.9991±0.0001  HD (mm)=9.626±2.064  ASD (mm)=0.008±0.003  - F: DSC=0.9890±0.0047  Sensitivity=0.9951±0.0019  Specificity=0.9991±0.0001  HD (mm)=3.897±2.526  ASD (mm)=0.009±0.004  V-Net  - F+M: DSC=0.9815±0.0009  Sensitivity=0.9906±0.0033  Specificity=0.9990±0.0000  HD (mm)=9.144±2.096  ASD (mm)=0.012±0.006  - M: DSC 0.9863±0.0012  Sensitivity=0.9865±0.0011  Specificity=0.9991±0.0001  HD (mm)=18.748±3.181  ASD (mm)=0.013±0.005  - F: DSC=0.9847±0.0009  Sensitivity=0.9827±0.0023  Specificity=0.9993±0.0001  HD (mm)=12.403±1.667  ASD (mm) = 0.015±0.025 | NR | NR | NR |
| [Patton et al., 2021](https://www.zotero.org/google-docs/?DUy7Rq) | DSC  - FN:  Otsu=0.445±0.130  Yen=0.894±0.056  Local Otsu=0.283±0.089  FN U-Net=0.966±0.018  VB U-Net=0.892±0.080  FN+VB U-Net=0.963±0.018 | NR | NR | NR |
| [Deng et al., 2022](https://www.zotero.org/google-docs/?ivzjG0) | Periosteal surface segmentation  DSC: 0.9782  ASD (mm): 0.1657  TNR: 0.9989  TPR: 0.9959  Endosteal surface segmentation  DSC: 0.9653  ASD (mm): 0.2764  TNR: 0.9990  TPR: 0.9915  FH (periosteal)  MAE (cm^3^): 1.2372  RMSE (cm^3^): 1.2809  RE (%) (min, mean, max): 1.81, 3.77, 6.75  FN (periosteal)  MAE (cm^3^): 0.2811  RMSE (cm^3^): 0.3102  RE (%) (min, mean, max): 0.89, 4.48, 6.67  Combination (periosteal)  MAE (cm^3^): 1.9800  RMSE (cm^3^): 2.1589  RE (%) (min, mean, max): 0.65, 3.18, 5.16  Cortical bone in FN  MAE (cm^3^): 0.0636  RMSE (cm^3^): 0.0934  RE (%) (min, mean, max): 0.18, 4.19, 14.10  Cortical bone in combination  MAE (cm^3^): 0.4643  RMSE (cm^3^): 0.5724  RE (%) (min, mean, max): 4.45, 3.15, 9.21  FN (endosteal)  MAE (cm^3^): 0.2462  RMSE (cm^3^): 0.2708  RE (%) (min, mean, max): 1.35, 4.82, 5.97  Combination (endosteal)  MAE (cm^3^): 2.03  RMSE (cm^3^): 2.23  RE (%) (min, mean, max): 1.52, 4.51, 5.79 | NR | NR | NR |
| [Zhang et al., 2022](https://www.zotero.org/google-docs/?LPAe9T) | DSC for each sample:  1) 0.9717, 2) 0.9768, 3) 0.9632, 4) 0.9746, 5) 0.9762  PA for each sample:  1) 0.9957, 2) 0.9983, 3) 0.9983, 4) 0.9982, 5) 0.9968  TPR for each sample:  1) 0.9492, 2) 0.9489, 3) 0.9384, 4) 0.9457, 5) 0.9439  TNR for each sample:  1) 0.9993, 2) 0.9995, 3) 0.9996, 4) 0.9989, 5) 0.9991 | NR | NR | NR |
| [Kuiper et al., 2022](https://www.zotero.org/google-docs/?750Y2P) | For femur: mean DSC 0.99, mean HD (mm) 2.5, mean MASD (mm) 0.1, mean HD95 (mm) 0.6  For best performing input configuration: DSC 0.971±0.013, HD (mm) 5.3±8.0, MASD (mm) 0.36±0.06, HD95 (mm) 0.87±0.12  (this is not specific for femur but considering the lower extremities) | MASD (mm) 0.58±0.07, HD (mm) 5.03 ±3.20 | NR | NR |
| [Bjornsson et al., 2023](https://www.zotero.org/google-docs/?WyeCrq) | Sample I: DSC=0.975±0.006  HD95 (mm) =1.04±0.33  vs  DSC =0.973±0.005  HD95 (mm)=1.06±0.16  using semi-automated method in [(Pauchard et al., 2016)](https://www.zotero.org/google-docs/?0cQPdQ)  Sample II:  DSC=0.990±0.008  HD95 (mm)=0.999±0.331  FE strength based on the proposed femur segmentation method compared to femoral strength based on manual femur segmentations:  - Left femur ⇒ R^2^ 0.986, RMSE (N) 212.2, MAE (%) -2.14, max difference (%) 25.3;  - Right femur ⇒ R^2^ 0.988, RMSE (N) 177.0, MAE (%) -1.86, max difference (%) 30.1 | Sample I: DSC=0.975±0.006  HD95 (mm) =1.04±0.33  Sample II:  DSC=0.990±0.008  HD95 (mm)=0.999±0.331 | NR | NR |
| [Apivanichkul et al., 2023](https://www.zotero.org/google-docs/?LMCT9M) | Highest DSC=0.8825 | NR | NR | NR |
| Tan et al., 2024 | DSC: 97.30 %  HD95 (pixel units): 6.27 | NR | NR | NR |
| Sultana et al., 2024 | Validated: DSC 0.992 ± 0.008, mean IoU 0.995 ± 0.005, average precision 0.996 ± 0.003, sensitivity 0.995 ± 0.004, specificity 0.998 ± 0.002  Unseen: DSC 0.918 ± 0.025, mean IoU 0.983 ± 0.016, average precision 0.923 ± 0.024, sensitivity 0.927 ± 0.02, specificity 0.999 ± 0.002 | NR | NR | NR |
| Zhang et al., 2024 | DSC (n = 20): 0.990 for femoral head FH, 0.941 for femoral neck FN and 0.981 for trochanteric region TR  vBMD measurements: CCC of 0.977 and a RMSCV (%) of 1.39% considering all patients; CCC of 0.986 and a RMSCV (%) of 1.07% considering non-obese patients; CCC of 0.947 and a RMSCV (%) of 2.06% considering obese patients. vBMD values using the two imaging protocols are correlated, ULD CT-based vBMD measures are higher than clinical CT-based values. | NR | NR | DSC for repeat scan reproducibility (n = 5) for the femoral subregions FH, FN, and TR were 0.982, 0.970, and 0.986, respectively.  vBMD measurements: CCC of 0.996 and a RMSCV (%) of 0.72% considering all patients; CCC of 0.998 and a RMSCV (%) of 0.51% considering non-obese patients; CCC of 0.995 and a RMSCV (%) of 0.83% considering obese patients. vBMD values using the two imaging protocols are correlated, ULD CT-based vBMD measures are higher than clinical CT-based values. |
| Saillard et al., 2024 | MEKANOS database:   - U-Net 2D multi axial (without pre-processing): DSC 0.74 ± 0.09, HD (mm) 49.86 ± 12.57; - U-Net 2D multi axial: DSC 0.93 ± 0.01, HD (mm) 2.30 ± 0.82; - U-Net 3D: DSC 0.96 ± 0.01, HD (mm) 2.20 ± 0.71; - nnUNet 3D fullres: DSC 0.96 ± 0.01, HD (mm) 2.40 ± 0.84   Secondary dataset:  - Shaft: DSC 0.979 ± 0.004, HD (mm) 1.69 ±10.37;  - Middle region: DSC 0.975 ±0.003, HD (mm) 2.77 ±20.71  - Head: DSC 0.976 ±0.003, HD (mm) 2.66 ±20.88  - Global: DSC 0.977 ±0.0022, HD (mm) 2.96 ±0.82  Absolute mean of difference with manual FE-based failure load estimates (N):  - 473 ± 336 with automatic segmentation + erosion (2 pixels)  - 168 ± 105 with automatic segmentation + erosion (1 pixel)  - 119 ± 66 with automatic segmentation  - 132 ± 121 with automatic segmentation + dilation (1 pixel)  - 203 ± 201with automatic segmentation + dilation (2 pixel)  Absolute mean of difference with manual FE-based failure load estimates (%):  7.42 ± 5.00 with automatic segmentation + erosion (2 pixels)  3.19 ± 2.55 with automatic segmentation + erosion (1 pixel)  2.25 ± 1.91 with automatic segmentation  2.79 ± 3.37 with automatic segmentation + dilation (1 pixel)  4.45 ± 5.42 with automatic segmentation + dilation (2 pixel) | NR | Mean DSC:  - Expert versus expert: 0.973 ± 0.013  - Expert vs Automatic: 0.980 ± 0.008  In terms of failure loads, the Authors report a bar plot showing the failure load values obtained on 6 ex-vivo femurs using different segmentations from 4 operators (beginner or expert) or automatic segmentation (Supplementary material) | NR |
| **Other methods** | | | | |
| [Testi et al., 2001](https://www.zotero.org/google-docs/?OHvmgR) | ME: 0.93, 0.77, 0.57 mm for manual, border-tracing and threshold-based methods, respectively.  ME only on diaphyseal region: 0.87, 0.66, and 0.58 mm for manual, border-tracing and threshold-based methods, respectively. | NR | Selection of 19 images from the in-vivo CT dataset, geometry extraction performed three times with border tracing method and threshold-based method, computing of distance between contour in terms of HD ⇒ decrease of RMSE of HD from 2.29 mm to 1.41 mm with border tracing method  Extraction of 5 images from the 6 in-vivo CT datasets of patients in need of a CMP, tracing of the contours three times with both methods, comparison between inner and outer contours in terms of HD ⇒ decrease of RMSE of HD from 5 to 1.5 mm with border tracing method | NR |
| [Kang et al., 2003](https://www.zotero.org/google-docs/?86JTuX) | L1: r_tot_ 18.15 mm (expected value) vs 18.58±0.11 (segmented images);  r_trab_ 17.32 mm (expected value) vs 17.19±0.09 (segmented images);  d_cort_ 0.83 mm (expected value) vs 1.39 ±0.13 (segmented images)  L2: r_tot_ 18.03 mm (expected value) vs 18.36 ±0.11 (segmented images);  r_trab_ 16.95 mm (expected value) vs 16.75 ± 0.13 (segmented images);  d_cort_ 1.09 mm (expected value) vs 1.61±0.13 (segmented images)  L3: r_tot_ 18 mm (expected value) vs 18.17±0.09 (segmented images);  r_trab_ 16.49 mm (expected value) vs 6.37±0.09 (segmented images);  d_cort_ 1.51 mm (expected value) vs 1.80±0.12 (segmented images) | An increase of the noise causes an increase of the segmented periosteal and endosteal volumes and the effect of an increase of noise is more pronounced when the cortical thickness is smaller | Intra-observer variability ⇒ CV_RMS_ (%): total volume 0.29±0.17, trabecular volume 0.64±0.37, CTh 1.54±1.10  Inter-observer variability ⇒ CV_RMS_ (%): total volume 0.27±0.15, trabecular volume 0.73±0.43, CTh 1.71±1.10 | NR |
| [Gelaude et al., 2008](https://www.zotero.org/google-docs/?riMMwJ) | For dry femurs (without soft tissues) comparison with respect to ground truths $\underline{X}$±SD (mm): -0.65±0.31 for entire femur, -0.65±0.30 for proximal part, -0.68±0.23 diaphyseal part and -0.62±0.40 for distal part  Comparison between dry femurs and femurs with soft tissues: 0.5 mm in terms of absolute difference  For femurs with soft tissues comparison with respect ground truths $\underline{X}$±SD (mm): -0.62±0.49 for entire femur, -0.67±0.45 for proximal part, -0.62±0.35 for diaphyseal part, -0.55±0.67 for distal part | NR | NR | NR |
| [O’Neill et al., 2012](https://www.zotero.org/google-docs/?B2x5C5) | VOE_g_ (%) 2.71±0.44  VOE_S_Avg (%) 3.30±0.56  VOE_S_min (%) 1.00±0.33  VOE_S_max (%) 36.84±25.17  SSD_gAvg_ (mm) 0.28 ±0.04  SSD_sAvg_Avg (mm) 0.29±0.04  SSD_Max_ (mm) 4.00±1.60 | NR | NR | NR |
| [Zou et al., 2017](https://www.zotero.org/google-docs/?4KuRMj) | JAC (%): 84.02±10.95 for proposed method, 81.01±11.93 for MS, 83.75±12.82 for LS and 83.29±12.23 for SPGC  DSC (%): 85.96±11.18 for proposed method, 83.39±12.36 for MS, 84.79±13.10 for LS and 81.84±10.30 for SPGC  DCD (mm): 0.52±0.19 for proposed method, 0.57±0.24 for MS, 0.54±0.24 for LS and 0.56±0.20 for SPGC | NR | NR | NR |
| [Gangwar et al., 2018](https://www.zotero.org/google-docs/?1r4dqf) | DSC: 0.9339±0.0287  TPR: 0.9339±0.0287  TNR: 0.9855±0.0115 | NR | NR | NR |
| [Väänänen et al., 2019](https://www.zotero.org/google-docs/?75CNba) | 13 in-vivo CT datasets  - DSC: whole femur 0.93±0.02, trabecular 0.91±0.02  - ASD (mm): whole femur 0.99±0.23, trabecular 0.99±0.20  - VD (cm^3^): whole femur −16.5±5.8, trabecular −8.9±5.8  The automatically generated FE models predicted femoral strains with high correlation (R^2^=0.89, NRMSE=6%, slope=0.96, intercept=121 μ$\epsilon$), although slightly lower than that obtained by manually generated models (R^2^=0.94, NRMSE=9%, slope=0.96, intercept=133 μ$\epsilon$) | 13 in-vivo CT datasets  - DSC: whole femur 0.93±0.02, trabecular 0.91±0.02  - ASD (mm): whole femur 0.99±0.23, trabecular 0.99±0.20  - VD (cm^3^): whole femur −16.5±5.8, trabecular −8.9±5.8  14 ex-vivo CT datasets  - DSC: whole femur 0.98±0.01, trabecular 0.97±0.01;  - ASD (mm): whole femur 0.21±0.07, trabecular 0.35±0.05  - VD (cm^3^): whole femur −1.68±2.99, trabecular −1.43±1.82 | NR | NR |
